# Neutrophil extracellular traps promote proliferation of pulmonary smooth muscle cells mediated by CCDC25 in pulmonary arterial hypertension

**DOI:** 10.1186/s12931-024-02813-2

**Published:** 2024-04-25

**Authors:** Hongxiao Sun, Zhanhui Du, Xu Zhang, Shuai Gao, Zhixian Ji, Gang Luo, Silin Pan

**Affiliations:** Heart Center, Women and Children’s Hospital, Qingdao University, Qingdao, China

**Keywords:** Coiled-coil domain containing 25, Neutrophil extracellular traps, Pulmonary arterial hypertension, Pulmonary artery smooth muscle cells

## Abstract

**Background:**

Previous studies have indicated that neutrophil extracellular traps (NETs) play a pivotal role in pathogenesis of pulmonary arterial hypertension (PAH). However, the specific mechanism underlying the impact of NETs on pulmonary artery smooth muscle cells (PASMCs) has not been determined. The objective of this study was to elucidate underlying mechanisms through which NETs contribute to progression of PAH.

**Methods:**

Bioinformatics analysis was employed in this study to screen for potential molecules and mechanisms associated with occurrence and development of PAH. These findings were subsequently validated in human samples, coiled-coil domain containing 25 (CCDC25) knockdown PASMCs, as well as monocrotaline-induced PAH rat model.

**Results:**

NETs promoted proliferation of PASMCs, thereby facilitating pathogenesis of PAH. This phenomenon was mediated by the activation of transmembrane receptor CCDC25 on PASMCs, which subsequently activated ILK/β-parvin/RAC1 pathway. Consequently, cytoskeletal remodeling and phenotypic transformation occur in PASMCs. Furthermore, the level of NETs could serve as an indicator of PAH severity and as potential therapeutic target for alleviating PAH.

**Conclusion:**

This study elucidated the involvement of NETs in pathogenesis of PAH through their influence on the function of PASMCs, thereby highlighting their potential as promising targets for the evaluation and treatment of PAH.

**Supplementary Information:**

The online version contains supplementary material available at 10.1186/s12931-024-02813-2.

## Background

Pulmonary arterial hypertension (PAH) is a common clinical disease that affects at least 1% of the global population [1, 2]. PAH is a heterogeneous clinical disease characterized by abnormally elevated pulmonary arterial pressure, which is characterized by pathological remodeling and vasoconstriction of pulmonary vessels, leading to progressive dyspnea, exercise tolerance, right ventricular failure, and death, diagnosis and treatment are hot topics in the field of PAH [3, 4].

The molecular mechanism of PAH is complex and multifactorial, mainly reflected in: inflammation, metabolic changes, and genetic or epigenetic abnormalities [5]. PAH results from the interaction of multiple cell types, including pulmonary artery endothelial cells (PAECs), pulmonary artery smooth muscle cells (PASMCs), fibroblasts, and various immune and circulating cells [5]. Pulmonary artery endothelial dysfunction leads to decreased vasodilator factor production and increased precontractual factor synthesis [6]. Growth factors and proinflammatory cytokines can stimulate the proliferation of PASMCs and extracellular matrix deposition and remodeling [7]. Abnormalities in transforming growth factor β (TGF β) and bone morphogenetic receptor protein 2 (BMPR2) are key drivers of PAH [8]. BMPR2 mutation and increased activity of the TGF β pathway promotes the abnormal proliferation of PAECs and PASMCs in PAH [4, 9]. Abnormal proliferation and phenotypic transformation of PASMCs play a crucial role in pathological changes observed in PAH [4, 10, 11]. Cytoskeletal remodeling of PASMCs plays a pivotal role in abnormal proliferation and phenotypic transformation [12].

Inflammation is a common phenomenon in PAH, and strong immune responses can be observed in many types of PAH [13–15]. BMPR2 can increase the expression of pro-inflammatory factors and promote the proliferation, migration and differentiation of PAECs, PASMCs and immune cells [13, 16, 17]. Autoantibodies and local lymphoid follicles are also involved in inflammation and immune activation in pulmonary hypertension [18, 19]. A variety of immune cells are also involved in the development of PAH. An inflammatory response surrounding pulmonary artery is evident in patients with PAH, and inhibition of this response can effectively suppress abnormal proliferation of PASMCs [20–22]. The significant involvement of neutrophils in PAH has been increasingly acknowledged [21, 22]. The upregulation of neutrophil extracellular traps (NETs) has been confirmed in both lung tissue and plasma samples obtained from patients with PAH [23]. However, the specific role of NETs in PAH, particularly impact on abnormal proliferation of PASMCs, has not been determined. CCL1, CCL2, CCL5 can recruit monocytes to migrate to the inflammatory site and play a role in the proliferation and dedifferentiation of PASMCs [13, 24, 25]. Patients with severe idiopathic pulmonary arterial hypertension have increased infiltration of macrophages and T and B lymphocytes in the pulmonary vessel wall, which promotes vasoconstriction and pulmonary vascular remodeling [13, 25, 26].

Remodeling due to metabolic disorders is an important driver of the pathogenesis of PAH [27]. Studies have shown that the cytoplasmic glycolytic pathway is upregulated in PAECs and PASMCs from patients with PAH [28–30]. Mitochondrial abundance, mitochondrial DNA and oxygen consumption in PAECs is reduced [28, 30]. Disruption of the mitochondrial network can be seen in PASMCs, as well as a reduction in mitochondrial respiration [31]. An increase in pyruvate dehydrogenase inhibitory kinase activity shown in PAH [32, 33]. A decrease in fatty acid oxidation mediators in cultured PAECs in vitro, but another study highlighted an increase in long-chain and medium-chain free fatty acid products [29, 34]. Glutamate metabolism has been found to be upregulated in the lung and right ventricle of patients with PAH, a finding that correlates with the proliferative phenotype [35–38].

BMPR2, EIF2AK4, TBX4 and other gene mutations may have genetic and epigenetic effects on PAH, among them, mutations in BMPR2 are the most common [39, 40]. Epigenetic modifications affect PAH through processes such as DNA methylation, acetylation, histone modification, and RNA-based modification [41].

NETs, which are generated through the process of neutrophil genesis NETosis, have been extensively investigated as pathogenic agents [23, 24]. Aldabbous et al. [23] demonstrated a significant increase in the expression of markers associated with NETs in the plasma and lung tissue of patients with PAH. Yang et. al [42] found that, the transmembrane protein coiled-coil domain containing 25 (CCDC25) functions as a receptor for NETs-DNA on the surface of cancer cells, activating the ILK/β-parvin/RAC1 pathway and enhancing cancer cell motility by sensing extracellular NETs-DNA. Sensing NETs-DNA is the only function of CCDC25 that has been identified so far [42–44].

Based on the above background, it is of great significance to explore the potential molecular mechanism of the pathogenesis of PAH. Inflammatory response plays an important role in PAH, and neutrophils are the most important and abundant immune cells. Therefore, we attempted to explore the role of NETs in the development and progression of PAH. In present study, we investigated the impact of NETs on PASMCs through CCDC25. Activation of ILK/β-parvin/RAC1 pathway induces cytoskeletal remodeling and phenotypic transformation in PASMCs, thereby promoting abnormal proliferation. This study underscores the potential significance of targeted NETs for both assessment and therapeutic interventions in PAH patients.

## Methods

### Bioinformatics data download and analysis

Bioinformatics data were obtained from GEO database (https://www.ncbi.nlm.nih.gov/geo/). Three microarray datasets, GSE53408 [45], GSE113439 [46], GSE117261 [47], and one single-cell sequence dataset GSE210248 [48] were collected. For microarray data, GEOquery [49], oligo [50] and sva [51] packages were used to retrieve, integrate, and eliminate batch effects from data. Limma [52] package was used for differential expression analysis, while hugene10sttranscriptcluster.db was used for gene annotation. Additionally, clusterProfiler [53] package was used to conduct enrichment analysis. Seurat [54] package was used for analysis of single-cell sequence data. Cell annotation was performed using cellmarker 2.0 website (http://117.50.127.228/CellMarker/). The methods employed in differential expression analysis and enrichment analysis were consistent with those used in analysis of microarray data.

### Clinical sample collection and processing

Peripheral blood samples were obtained from children with or without PAH. The inclusion criteria for children with PAH were those who met the diagnostic criteria for PAH and had PAH as their first diagnosis without other diseases [55]. Control group were healthy volunteers. Subsequently, samples were allowed to stand at room temperature for 30 min, followed by centrifugation at 300 × g for 10 min. The resulting supernatant was collected and stored at -80℃. Moreover, mean pulmonary arterial pressure (mPAP), which was determined using echocardiography, was used to assess severity of PAH [1, 56].

### Cell-free DNA(cf-DNA)detection

A Quant-It Picogreen dsDNA assay kit (Thermo Fisher Scientific Inc, USA) was used to determine concentrations of cf-DNA. Serum samples were diluted 10-fold and processed on 96-well plates following instruction. Standard curves were generated using ELISA Clac (v0.2, BLUE GENE, China) with a linear regression equation model.

### Enzyme-linked immunosorbent assay (ELISA)

Concentrations of myeloperoxidase (MPO) and neutrophil elastase (NE) were quantified using a Human Myeloperoxidase ELISA Kit (Abcam, UK) and a Human PMN Elastase ELISA Kit (Abcam, UK), respectively. Following a 10-fold dilution of serum samples, the assays were performed according to instructions. Standard curves were generated using an ELISA Clac (v0.2, BLUE GENE, China) employing a five-parameter logistic regression equation model.

### Purification of NETs

NETs utilized in this study were derived from peripheral blood neutrophils of normal Sprague‒Dawley (SD) rats. Neutrophils were isolated using a Rat Neutrophil Isolation Kit (Solarbio, China). Subsequently, NETs were generated by stimulating neutrophils with 20 nmol/L phorbol 12-myristate 13-acetate (PMA, Sigma Aldrich, USA) for 12 h [23]. To collect NETs, the bottom of culture bottle was vigorously rinsed and then centrifuged at 300 × g for 10 min to obtain supernatant containing NETs. The concentration of cf-DNA served as a quantitative indicator of concentration of NETs.

### Cell culture, CCDC25 knockdown and grouping

Primary PASMCs were isolated from small pulmonary arteries of normal SD rats using tissue patch method as previously described [57]. PASMCs were cultured in complete DMEM/F12 medium (Pricella, China) supplemented with 10% fetal bovine serum (Pricella, China) and 100 µg/mL penicillin‒streptomycin (Pricella, China), at 37 °C in a humidified incubator with 5% CO_2_.

PASMCs were transfected with lentiviral-based shRNA targeting CCDC25 to downregulate its expression. The shRNA construct targeting CCDC25 (shCCDC25) was designed and provided by Genechem Co., Ltd. Knockdown vector used was GV493, which contained hU6-MCS-CBh-gcGFP-IRES-puromycin sequence. DNA template oligonucleotides of three distinct shCCDC25 sequences, and one control sequence were generated as outlined below:

shCCDC25-1: CCTCAAATCAAGATGGTAACG.

shCCDC25-2: GCAGAAGGATGTAAAGATTGT.

shCCDC25-3: GAGAGAAGATAGAAGACATTC.

Control: TTCTCCGAACGTGTCACGT.

CCDC25 knockdown procedure was conducted according to manufacturer’s instruction, and the efficiency of knockdown procedure was verified via fluorescence microscopy and western blotting.

Concentration of NETs used in this study was 0.3 µg/mL [23], while positive control was established using recombinant human platelet-derived growth factor-BB (PDGF-BB; Sangon Biotech, China) at a concentration of 20 µg/L [58]. Intervention duration was 24 h. Normal: PASMCs without treatment; Control: scrambled shRNA intervention; shCCDC25: PASMCs transfected with shCCDC25.

PASMCs were divided into the following groups: (1) Normal, Normal + NETs, and Normal + PDGF-BB; (2) Control, Control + NETs, Control + PDGF-BB; and (3) shCCDC25, shCCDC25 + NETs, and shCCDC25 + PDGF-BB.

### Cell counting kit − 8 (CCK-8) assay

PASMCs were seeded in 96-well plates at a density of 1,000 cells per well and cultured for 24 h under different experimental conditions. Following incubation, CCK-8 mixture (Yeasen, China) was added to each well (10 µL per well). Then PASMCs were further incubated for an additional hour in a cell incubator, after which optical density at 450 nm was measured.

### 5-Ethynyl-2’-deoxyuridine (EdU) assay

PASMCs were seeded in 96-well plates at a density of 5,000 cells per well and cultured for 24 h following different treatments. EdU assays were performed as instructed by YF®594 Click-iT EdU Imaging Kits (Yeasen, China). Fluorescence microscopy images were acquired and cell counts were determined using ImageJ software (v1.53, NIH, USA).

### Flow cytometry

PASMCs were seeded in 6-well plates at a density of 500,000 cells per well and cultured for 24 h following different treatments. Flow cytometry analysis was performed using a Cell Cycle and Apoptosis Analysis Kit (Yeasen, China). Results were analyzed using FlowJo software (v10.8.1., BD Biosciences, USA).

### Western blotting

Proteins were extracted from tissues or cells with RIPA buffer (Epizyme, China). Total protein concentration was determined using a protein concentration detector. Proteins were separated by SDS-PAGE (Epizyme, China), transferred onto PVDF membranes (Millipore, Germany), and blocked with a solution of skim milk powder at a concentration of 5% for two hours. The target proteins were probed with corresponding primary antibodies at 4 °C overnight, and then washed with TBST followed by incubation with an HRP-conjugated secondary antibody at room temperature for one hour. Finally, the bands on membranes were visualized through chemiluminescence, and gray value analysis was performed using ImageJ software (v1.53, NIH, USA). Manufacturers and catalogs of antibodies used were listed in additional file 1: Table S1.

### Cytoskeleton staining

PASMCs were seeded in 24-well plates at a density of 50,000 cells per well and cultured for 24 h. Cytoskeleton was stained with Actin-Tracker Red-594 (Beyotime, China) according to manufacturer’s instruction. Fluorescence microscopy was used to capture images.

### Animal experiments

Eight-week-old male SD rats were selected, and a PAH model was induced by intraperitoneal injection of monocrotaline (MCT, MedchemExpress, USA) at a dose of 60 mg/kg after one week of acclimatization. To disrupt NETs, DNase I (Sigma‒Aldrich, Germany) was injected into the tail vein at week one and two weeks post modeling at dosage of 10 mg/kg [59]. A total of twenty-four SD rats were randomly divided into four groups: Normal group, MCT group, MCT + DNase I group, and Normal + DNase I group. Rat weights were measured every three days throughout experimental period, and the result was conducted by using repeated measures ANOVA. After four weeks, rats were euthanized to collect peripheral blood serum as well as lung and heart tissue samples for further analysis. Concentration of cf-DNA in peripheral blood serum was measured, and right ventricular hypertrophy index [RVHI = weight of right ventricle/(weight of left ventricle + ventricular septum)] was calculated. Lung tissue sections were stained with hematoxylin and eosin (H&E) for histopathological examination, while western blotting analysis was performed on proteins extracted from lung tissues.

### H&E staining

Paraffin sections were prepared following fixation, dehydration, and embedding in paraffin for subsequent analysis via H&E staining. H&E staining was performed using Hematoxylin and Eosin Staining Kit (Yeasen, China), according to manufacturer’s instruction. For each animal sample, 5 pulmonary arterioles (100–150 μm) were selected for quantitative analysis. Sildeveiwer software was used to calculate the percentage of vascular wall thickness (WT)%=2MT/ED×100% (MT, media thickness; ED, external diameter); Percentage of vascular wall area (WA)%= (TA-IA)/TA×100% (TA, mean total vessel area; IA, vascular lumen area).

### Statistical analysis

Statistical analysis was performed using SPSS Statistics (v26, IBM, USA). Descriptive statistics were used to summarize all variables in terms of mean ± standard deviation. Appropriate statistical method was selected based on nature of data. The number of replicates and additional statistical details are provided in figure legends. Statistical significance was determined at a threshold of *p* < 0.05.

## Results

### Microarray analysis revealed NETs and cytoskeletal changes in PAH

**Fig. 1:**
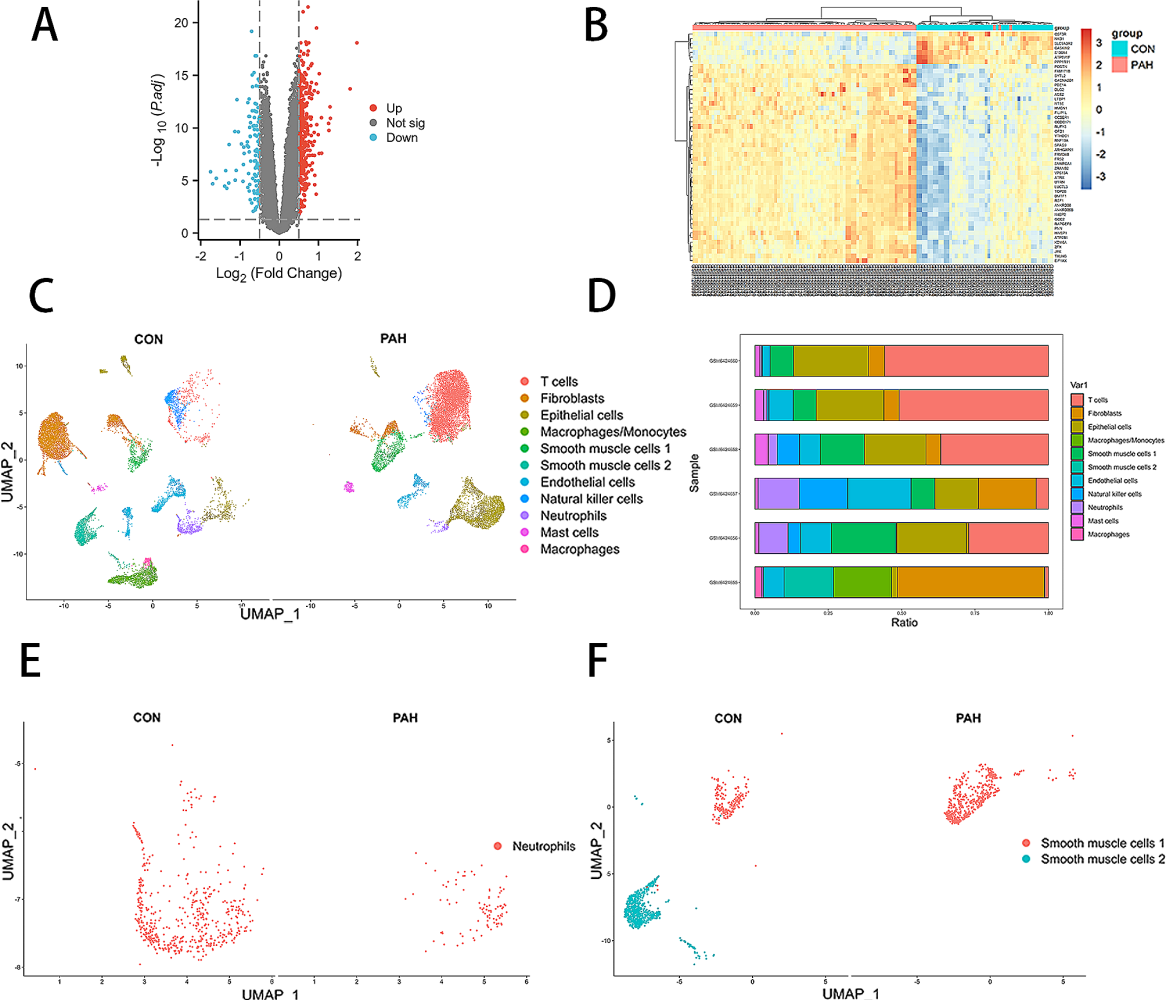
Bioinformatics analysis of patients with or without PAH. (**A**) Microarray data difference analysis volcano map. (**B**) Clustering heatmap of the top 50 differential genes in the microarray data. (**C**) Single-cell sequencing data, subpopulation grouping UMAP. (**D**) Single-cell sequencing data, cell proportion map of each sample. (**E**) Single-cell sequencing data, neutrophil grouping UMAP. (**F**) Single-cell sequencing data, PASMCs grouping UMAP

To identify molecules and molecular mechanisms related to PAH, bioinformatics analysis was conducted on publicly available PAH-related human lung tissue data. Differential expression analysis of DNA microarray data revealed a total of 18,857 genes, including 370 upregulated genes and 115 downregulated genes among the set of 485 differentially expressed genes (DEGs). Volcano plots were generated for all DEGs (Fig. 1A), while cluster heatmaps were created for the top 50 genes with the smallest adjusted *p* values (Fig. 1B).

A total of 49 entries were obtained from GO and KEGG enrichment analysis. GO results revealed significant alterations in inflammation, extracellular structures and DNA conformation in lung tissue in patients with PAH compared to those in normal individuals (Additional file 2: Fig. S1A-C). Furthermore, KEGG analysis demonstrated vascular smooth muscle contraction in lung tissue in PAH patients (Additional file 2: Fig. S1D). These findings suggest that specific inflammatory extracellular structures might exert an impact on PASMCs in individuals with PAH, thereby prompting subsequent GSEA.

GSEA analysis results demonstrated neutrophil changes, dysregulation of neutrophil extracellular trap formation in lung tissue from PAH patients (Additional file 3: Fig. S2A-F). Notably, through comprehensive GSEA enrichment analysis, we enriched biological processes related to the cytoskeleton, including, cytoskeleton-dependent intracellular transport, regulation of microtubule cytoskeleton organization, cytoskeleton-dependent cytokinesis, protein localization to microtubule cytoskeleton, protein localization to cytoskeleton, regulation of cytoskeleton organization, microtubule cytoskeleton organization involved in mitosis, microtubule cytoskeleton regulation and regulation of actin cytoskeleton (Additional file 3: Fig. S2G-L). These finding indicated that NETs and cytoskeletal play roles in PAH.

### Single-cell sequence data revealed abnormal changes in neutrophils and PASMCs

Based on the microarray data, we focused on neutrophils and smooth muscle cells, and then analyzed the single-cell sequencing data to further explore these two cells. By analyzing a total of 22,704 pulmonary artery cells (11,759 normal cells and 10,945 PAH cells) from single-cell sequencing data, a comprehensive set of 11 distinct cell subsets was identified. These subsets included one neutrophil subset and two smooth muscle subsets (Fig. 1C). Additionally, proportionally stacked histograms were generated to visualize distribution of different cell types (Fig. 1D). Neutrophil subset (Fig. 1E), as well as two distinct populations of smooth muscle cells (Fig. 1F) was specifically isolated for further analysis. Subsequently, differential expression analysis was performed within neutrophil subsets across subgroups resulting in identification of 115 DEGs out of 19,662 genes. Among these DEGs, 56 genes were upregulated and 59 genes were downregulated. Furthermore, a comparison between two populations of smooth muscle cells revealed a total of 494 DEGs out of 19,662 genes analyzed, including 212 upregulated genes and 282 downregulated genes.

A total of 239 entries were obtained from GO and KEGG enrichment analysis based on DEGs in neutrophils. The top 10 entries with the smallest adjusted *p* values were plotted. Compared to those in normal samples, major histocompatibility complex expression and neutrophil degranulation were altered in patients with PAH (Additional file 4: Fig. S3A-D). Additionally, GSEA-KEGG results indicated occurrence of neutrophil death in PAH patients (Additional file 4: Fig. S3E). These findings collectively suggested that NETs formation might occur in neutrophils.

A total of 540 entries were obtained from GO and KEGG enrichment analysis based on DEGs in smooth muscle cells. The top 10 entries with the smallest adjusted *p* values were plotted. Results of the enrichment analysis revealed significant alterations in cellular metabolism (Additional file 5: Fig. S4A-D). GSEA demonstrated that cytoskeletal regulation was enriched (Additional file 5: Fig. S4J). These findings suggested a potential phenotypic transition from contractile to secretory state in smooth muscle cells.

### Level of NETs in peripheral blood serum of patients with PAH was increased

**Fig. 2:**
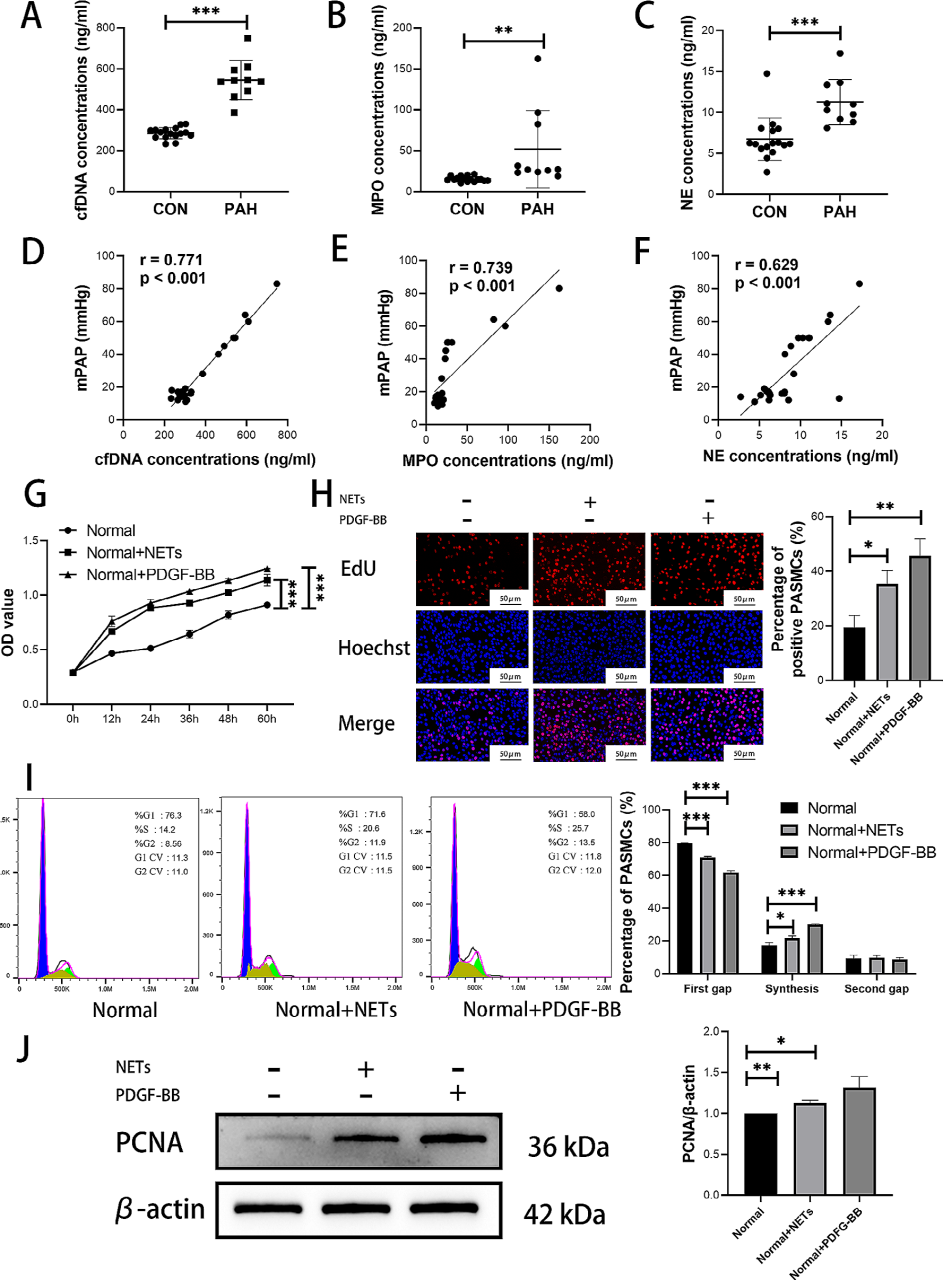
NETs was involved in the development of PAH. (**A-C**) Concentrations of cf-DNA, MPO and NE in human peripheral blood serum. (**D-F**) Correlation between NETs concentration and mPAP. (**G**) CCK-8 assay to detect the effect of NETs on PASMCs proliferation. (**H**) EdU to detect the effect of NETs on PASMCs proliferation. (**I**) Flow cytometry to detect the effect of NETs on PASMCs cell cycle. (**J**) Western blotting analysis of proliferation related PCNA in PASMCs. Protein levels were normalized to β-actin. All results were presented as the mean ± SEM (*n* = 3). The *p* values were determined by Student’s t-test. NS: *p* > 0.05, * *p* < 0.05, ** *p* < 0.01, *** *p* < 0.001

To investigate clinical significance of NETs in patients with PAH, concentrations of NETs markers were compared between PAH patients and non-PAH patients, and clinical correlation was analyzed. Peripheral blood samples were collected from a total of 26 children—10 with PAH and 16 without PAH. All samples were detected to determine the levels of cf-DNA, MPO, and NE. The results revealed that levels of cf-DNA, MPO, and NE were greater in PAH patients than in non-PAH individuals (Fig. 2A-C; Table 1). Furthermore, positive correlations between these three markers and mPAP within the studied patient population were observed (Fig. 2D-F).

**Table 1:** Statistics of clinical sample results

	CON		PAH		*P* value
Sex	Female	Male	Female	Male	0.28
	10	6	4	6	
Age (year)	1.15 ± 1.48		2.65 ± 2.43		0.09
cf-DNA (ng/ml)	285.25 ± 27.76		545.44 ± 95.80		**< 0.001**
MPO (ng/ml)	15.45 ± 3.21		51.87 ± 47.37		**0.004**
NE (ng/ml)	6.75 ± 2.58		11.26 ± 2.77		**< 0.001**
mPAP (mmHg)	15.19 ± 2.29		52.00 ± 14.73		**< 0.001**

### NETs can promote PASMCs proliferation in vitro

As proliferation of PASMCs plays a crucial role in development of PAH, the impact of NETs on PASMCs proliferation was investigated. CCK-8 assays were performed at six time points (0 h, 12 h, 24 h, 36 h, 48 h, and 60 h) following NETs intervention in PASMCs. The results demonstrated that NETs significantly enhanced PASMCs proliferation (Fig. 2G). Additionally, after exposure to NETs, PASMCs were subjected to EdU, which revealed a substantial increase in the number of proliferative cells compared that in normal PASMCs (Fig. 2H). Flow cytometry analysis was used to assess cell cycle alterations in PASMCs groups treated with or without NET. The findings indicated an increased proportion of PASMCs entering the division phase as G1 phase decreased and S phase increased upon treatment with NETs (Fig. 2I). Furthermore, Western blotting revealed upregulated protein expression levels of PCNA, a marker of cellular proliferation, in PASMCS cells exposed to NETs (Fig. 2J). Collectively, these results strongly suggested that NETs contributed to enhanced proliferation of PASMCs.

### NETs can promote cytoskeletal remodeling and phenotypic transformation of PASMCs via ILK/β-parvin/RAC1 pathway in vitro

**Fig. 3:**
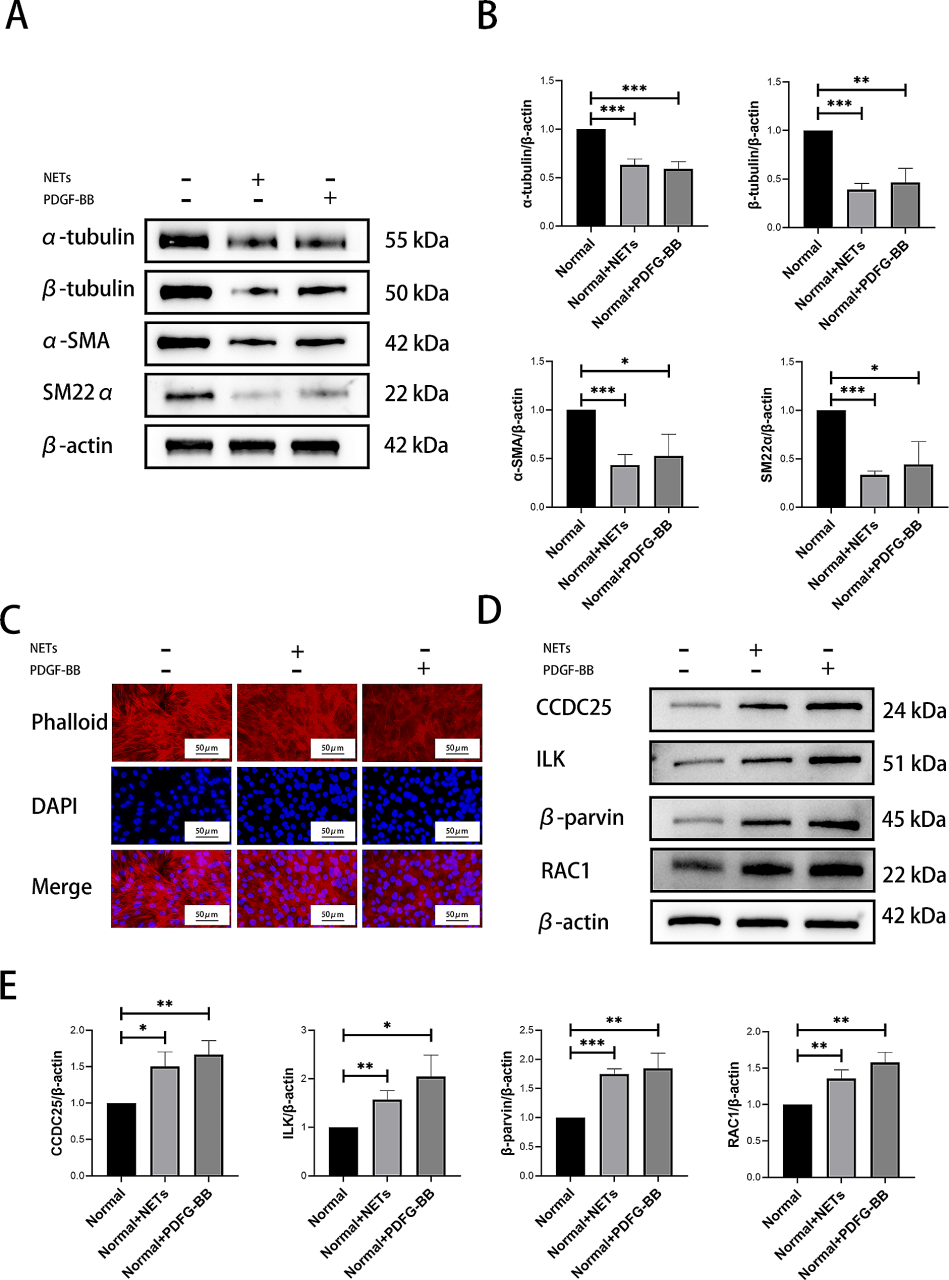
NETs can promote cytoskeletal remodeling and phenotypic transformation of PASMCs via ILK/β-parvin/RAC1 pathway in vitro. (**A-B**) Western blotting analysis of cytoskeletal related α-tubulin, β-tubulin, α-SMA, and SM22α in PASMCs. (**C**) Phalloidin staining to evaluate the effect of NETs on the cytoskeleton of PASMCs. (**D-E**) Western blotting analysis of CCDC25, and ILK/β-parvin/RAC1 pathway in PASMCs. Protein levels were normalized to β-actin. All results were presented as the mean ± SEM (*n* = 3). The *p* values were determined by Student’s t-test. NS: *p* > 0.05, * *p* < 0.05, ** *p* < 0.01, *** *p* < 0.001

Bioinformatics analysis revealed cytoskeletal changes in lung tissues of PAH patients. Phenotypic transformation is a characteristic phenomenon of PASMCs proliferation. Subsequent experiments to assess impact of NETs on cytoskeletal remodeling and phenotypic transformation in PASMCs were also conducted. Western blotting results revealed downregulation of α-tubulin, β-tubulin (cytoskeleton-related proteins), α-SMA, and SM22α (phenotypic transformation-related proteins) following NETs intervention (Fig. 3A-B). Additionally, cytoskeleton staining demonstrated a disordered distribution in NETs intervention group compared to normal group (Fig. 3C). The expression of CCDC25, a downstream receptor protein of NETs, was increased in NETs intervention group (Fig. 3D-E). Furthermore, key components of cytoskeleton-related pathways such as ILK, β-parvin and RAC1 were upregulated in NETs intervention group (Fig. 3D-E). These findings suggested that by stimulating PASMCs with CCDC25, NETs might activate ILK/β-parvin/RAC1 pathway leading to cytoskeletal remodeling and phenotypic transformation to promote PASMCs proliferation.

### Level of NETs in rats with PAH was increased and DNase I can alleviates PAH by disrupting NETs

**Fig. 4:**
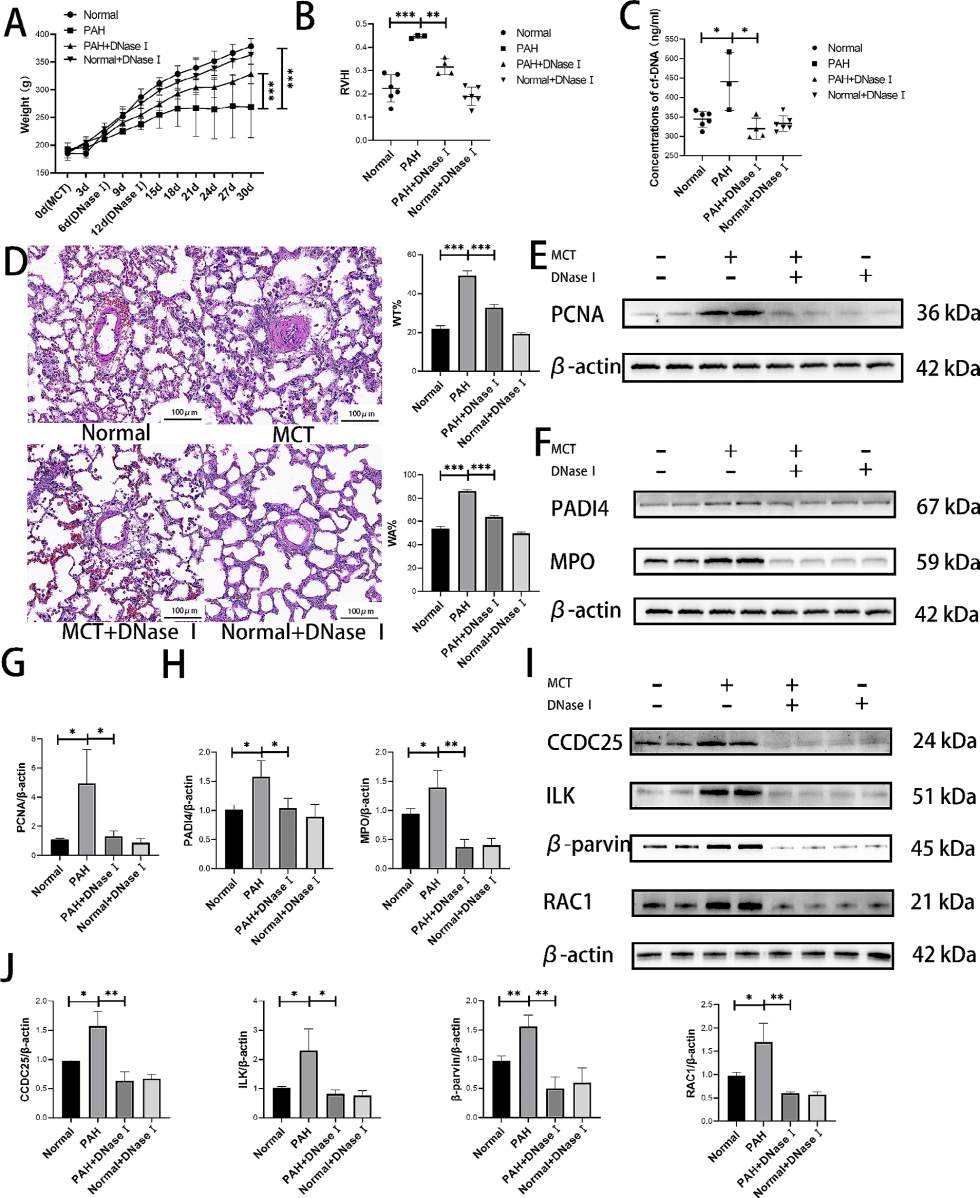
Removal of NETs in rats can inhibit the ILK/β-parvin/RAC1 pathway to relieve PAH. (**A**) Disrupting NETs can alleviate weight loss caused by MCT. (**B**) Disruption of NETs can alleviate right ventricular hypertrophy index caused by MCT. (**C**) Concentrations of cf-DNA in rats peripheral blood serum. (**D**) Disruption of NETs can alleviate pulmonary artery thickening caused by MCT. (**E**) Western blotting analysis of proliferation related PCNA in pulmonary tissue. (**F**) Western blotting analysis of NETs-related PADI4 and MPO in pulmonary tissue. (**G**) Statistical plot of PCNA western blotting results. (**H**) Statistical plot of NETs-related PADI4 and MPO western blotting results. (**I-J**) Western blotting analysis of CCDC25, and ILK/β-parvin/RAC1 pathway in pulmonary tissue. Protein levels were normalized to β-actin. All results were presented as the mean ± SEM (*n* = 3). The *p* values were determined by Student’s t-test. NS: *p* > 0.05, * *p* < 0.05, ** *p* < 0.01, *** *p* < 0.001

To evaluate the functionality of NETs, an in vivo assessment was conducted. PAH was induced in rats using MCT, and DNase I, a chemical drug capable of disrupting cf-DNA, was used to disrupt NETs. During the modeling process, three rats in MCT group died, while two rats in MCT + DNase I group died. Analysis of body weight revealed a significant decrease in MCT group, which subsequently increased following destruction of NETs by DNase I treatment (Fig. 4A). RVHI exhibited a significant increase in MCT group but decreased after treatment with DNase I (Fig. 4B). Concentrations of NETs in peripheral blood were greater in MCT group than in normal group but decreased upon addition of DNase I (Fig. 4C). H&E staining demonstrated notable thickening of pulmonary artery walls within MCT group, which was alleviated after DNase I treatment (Fig. 4D). Western blotting revealed significant upregulation of PCNA (Fig. 4E and G), and the NETs-related proteins PADI4 and MPO in MCT group (Fig. 4F and H), and these changes all showed subsequent decreases upon addition of DNase I. The expression of CCDC25 and ILK/β-parvin/RAC1 pathway related proteins was significantly inhibited following disruption of NETs (Fig. 4I-J).

### NETs can activate ILK/β-parvin/RAC1 pathway through CCDC25, promote cytoskeletal remodeling and phenotypic transformation of PASMCs, and lead to proliferation of PASMCs

**Fig. 5:**
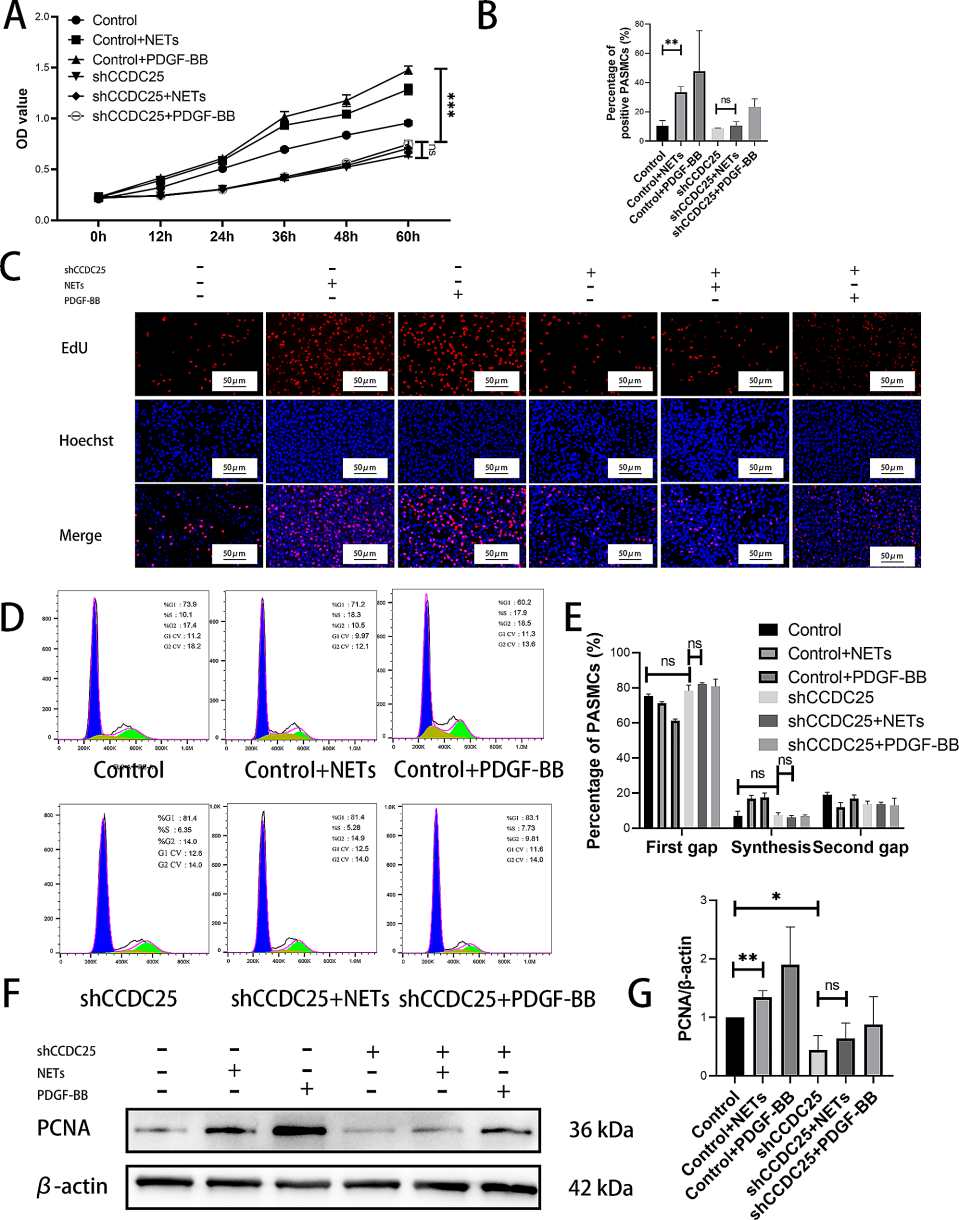
NETs affect PASMCs proliferation through CCDC25 in vitro. (**A**) CCK-8 assay to detect the effect of NETs and CCDC25 on PASMCs proliferation. (**B-C**) EdU to detect the effect of NETs and CCDC25 on PASMCs proliferation. (**D-E**) Flow cytometry to detect the effect of NETs and CCDC25 on PASMCs cell cycle. (**F-G**) Western blotting analysis of proliferation related PCNA in shCCDC25 PASMCs. Protein levels were normalized to β-actin. All results were presented as the mean ± SEM (*n* = 3). The *p* values were determined by Student’s t-test. NS: *p* > 0.05, * *p* < 0.05, ** *p* < 0.01, *** *p* < 0.001

**Fig. 6:**
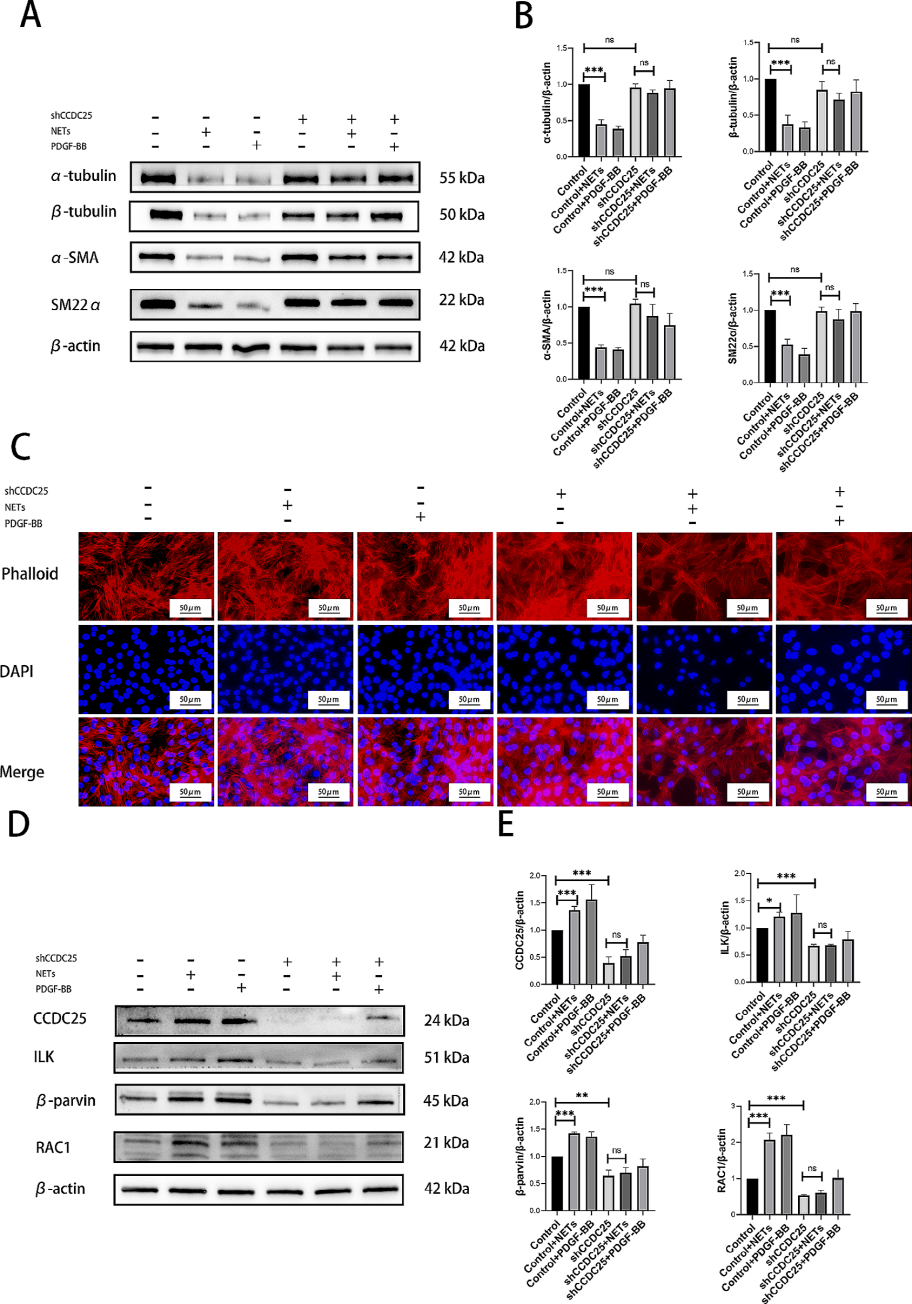
NETs can activate ILK/β-parvin/RAC1 pathways to promote cytoskeletal remodeling and phenotypic transformation of PASMCs through CCDC25 in vitro. (**A-B**) Western blotting analysis of cytoskeletal related α-tubulin, β-tubulin, α-SMA, and SM22α in shCCDC25PASMCs. (**C**) Phalloidin staining to evaluated the effect of NETs on the cytoskeleton of shCCDC25 PASMCs. (**D-E**) Western blotting analysis of CCDC25, and ILK/β-parvin/RAC1 pathway in shCCDC25 PASMCs. Protein levels were normalized to β-actin. All results were presented as the mean ± SEM (*n* = 3). The *p* values were determined by Student’s t-test. NS: *p* > 0.05, * *p* < 0.05, ** *p* < 0.01, *** *p* < 0.001

To confirm the role of CCDC25 in NETs, shCCDC25 PASMCs were generated. CCK-8 demonstrated that knockdown of CCDC25 inhibited proliferation of PASMCs, and even with the addition of NETs (Fig. 5A). EdU revealed a decrease in the number of proliferative cells after knockdown of CCDC25, and this trend persisted even when NETs were added (Fig. 5B-C). Cell cycle analysis revealed no significant changes in the proportion of cells in G1 phase or S phase after knockdown of CCDC25, and the addition of NETs did not alter these results (Fig. 5D-E). Western blotting indicated that PCNA expression decreased with knockdown of CCDC25 compared to that in control group, and there was no increase after the addition of NETs (Fig. 5F-G). Furthermore, the levels of α-tubulin, β-tubulin, α-SMA and SM22α did not change following knockdown of CCDC25, and were not reversed by the addition of NETs (Fig. 6A-B). Additionally, cytoskeletal structure was not affected by knockdown of CCDC25 even in the presence of NETs (Fig. 6C). The levels of proteins related to cytoskeleton-associated pathways such as ILK, β-parvin, and RAC1 decreased after knockdown of CCDC25 but did not increase after the addition of NETs (Fig. 6D-E).

## Discussion

The incidence of PAH is approximately 25 per million people, with an annual incidence of 2–5 per million people [60]. Several researchers have suggested that PAHs might affect around 1% of the global population and more than 10% of individuals aged 65 or older [1, 2]. Although current drug therapy and surgical interventions for PAH effectively alleviate symptoms and enhance life quality of patients, they do not provide a cure. Consequently, patients continue to bear the burden of long-term medication or face potential risks associated with surgical treatments.

The pathogenesis of PAH involves an inflammatory process. The specific role of neutrophils in PAH has not been fully elucidated, although mounting evidence suggests their significant involvement in the pathogenesis of this disease. The neutrophil/lymphocyte ratio (NLR) is elevated in PAH patients and has been shown to be significantly association with clinical deterioration and adverse outcomes [61–63]. Inhibition of NE could effectively alleviate PAH in both PAH patients and rat models by inducing PASMCs apoptosis [64, 65]. Furthermore, MPO can stimulate the proliferation of PASMCs through the Rho kinase pathway and reactive oxygen species pathway, leading to remodeling of the pulmonary vasculature, vascular remodeling is an important manifestation of PAH [66–68]. NETs, resulting from neutrophil NETosis, have been extensively investigated as a pathogenic factor [69, 70]. Aldabbous et al. [23] demonstrated a significant increase in the expression of markers associated with NETs in the plasma and lung tissue of patients with PAH. In the present study, elevated levels of cf-DNA, MPO, and NE in the serum of PAH patients were observed. Furthermore, these markers showed a positive correlation with mPAP. Additionally, animal experiments revealed increased concentrations of NETs markers in both serum and lung tissue samples from rats in the MCT group. Building upon our findings, evaluating the severity and progression of PAH by detecting NETs markers, such as cf-DNA from peripheral blood within a clinical setting is convenient and feasible. Abnormal proliferation and phenotypic transformation of PASMCs play a crucial role in pathological changes observed in PAH [4, 10, 11]. Existing studies have shown that NETs promote the proliferation of vascular smooth muscle cells through the Hippo-YAP pathway and Akt/CDKN1b/TK1 pathway in abdominal aortic aneurysm and hypertension, respectively [71, 72]. However, there is a lack of relevant studies on the effect of NETs on PASMCs in PAH. Therefore, we intervened PASMCs with NET. Results demonstrated a significant increase in the proliferative activity of PASMCs stimulated by NETs. Collectively, these results suggested that NETs could promote PASMCs proliferation, and may related to PAH.

DNase I can disrupt the cf-DNA mesh structure in NETs, which is the basis for NETs to function [70]. Previous reports have demonstrated the efficacy of DNase I in managing conditions such as cystic fibrosis [73, 74], acute respiratory distress syndrome [75], and refractory lobar atelectasis [76]. Interestingly, in the present study, the symptoms of PAH rats treated with DNase I were significantly alleviated, as indicated by weight recovery, alleviation of pulmonary vascular remodeling and inhibition of proliferative proteins. Considering the positive outcomes observed with DNase I administration in animal experiments conducted in this study, we propose that employing DNase I holds promise for treating PAH.

ILK/β-parvin/RAC1 pathway is a cytoskeleton-associated signaling cascade [77]. The interaction between ILK and β-parvin plays a pivotal role in regulating cell proliferation, migration, and cytoskeletal remodeling. Specifically, ILK facilitates the binding of β-parvin to α-actin, thereby promoting actin remodeling [78–80]. ILK forms complexes with β-parvin and pinches to regulate F-actin assembly, serving as mechanical signals that promote cytoskeletal reorganization and cytoskeleton-dependent cell adhesion processes [80]. ILK is involved in the regulation of actin dynamics by modulating small GTPases such as RhoA and RAC1 [81]. There is increasing evidence implicating the cytoskeletal remodeling and phenotypic transformation in vascular remodeling in PAH [82, 83]. Yang et al. [42] found that NETs could activate ILK/β-parvin/RAC1 pathway. Our study revealed significant downregulation of proteins associated with cytoskeletal remodeling and phenotypic transformation in PASMCs stimulated by NETs. Moreover, NETs were shown to activate ILK/β-parvin/RAC1 pathway in cell experiments. Additionally, animal experiments revealed upregulation of ILK/β-parvin/RAC1 pathway proteins in PAH rats. However, their expression was inhibited after destroying NETs. These results suggested that NETs activate ILK/β-parvin/RAC1 pathway, which leads to cytoskeletal remodeling and phenotypic transformation in PASMCs.

The transmembrane protein CCDC25 functions as a receptor for NETs-DNA on the surface of cancer cells, activating the ILK/β-parvin/RAC1 pathway and enhancing cancer cell motility by sensing extracellular NETs-DNA [42]. Subsequent investigations revealed that NETs could induce CCDC25 expression in gastric cancer cells, leading to increased proliferation of these cells [44]. Through animal experiments, an increase in CCDC25 expression was observed in MCT group, which was significantly decreased after NETs destruction. Cell-based experiments also demonstrated that NETs could stimulate CCDC25 expression in PASMCs, suggesting that NETs interact with CCDC25 in PASMCs. Knockdown of CCDC25 in PASMCs with shRNA blocked the association between NETs and PASMCs. Even if NETs were used to stimulate CCDC25-knockdown PASMCs, cytoskeletal remodeling and phenotypic transformation could not be activated, proliferation of PASMCs could not be stimulated, and ILK/β-parvin/RAC1 pathway could not be activated. These results indicated that CCDC25 is a key protein involved in the function of NETs in PASMCs and transmits stimulus signals into cells through this pathway.

## Conclusion

In summary, our findings from bioinformatics analysis, clinical samples, and animal and cell experiments demonstrated that NETs could activate ILK/β-parvin/RAC1 pathway in PASMCs via the membrane protein CCDC25. This activation led to cytoskeletal remodeling and phenotypic transformation of PASMCs, promoting the proliferation of PASMCs, and ultimately may relate to PAH. Importantly, our study unveiled a novel potential target for PAH diagnosis and treatment, offering hope for improved outcomes.

### Electronic supplementary material

Below is the link to the electronic supplementary material.

**Additional file 1: table S1**. Manufacturer and catalog of antibodies.

**Additional file 2: fig. S1**. GO and KEGG enrichment analysis for microarray data. (A-C) GO enrichment analysis results. (D) KEGG enrichment analysis results.

**Additional file 3: fig. S2**. GSEA for microarray data.

**Additional file 4: fig. S3**. Enrichment analysis and GSEA for neutrophil single-cell sequencing data. (A-C) GO enrichment analysis results for neutrophil. (D) KEGG enrichment analysis results for neutrophil. (E) GSEA results for neutrophil.

**Additional file 5: fig. S4**. Enrichment analysis and GSEA for PASMCs single-cell sequencing data. (A-C) GO enrichment analysis results for PASMCs. (D) KEGG enrichment analysis results for PASMCs. (E) GSEA results for PASMCs.

**Supplementary Material 6**

## Data Availability

No datasets were generated or analysed during the current study.

## Abbreviations

CCDC25: Coiled-coil domain containing 25
DEGs: Differential expression genes
MCT: Monocrotaline
mPAP: Mean pulmonary artery pressure
NETs: Neutrophil extracellular traps
NLR: Neutrophil/lymphocyte ratio
PAH: Pulmonary artery hypertension
PASMCs: Pulmonary artery smooth muscle cells
PDGF-BB: Human platelet-derived growth factor-bb
RVHI: Right ventricular hypertrophy index
