# Supplementary material for: Neutrophil extracellular traps promote proliferation of pulmonary smooth muscle cells mediated by CCDC25 in pulmonary arterial hypertension
Source: Respir Res. 2024 Apr 25;25:183. doi: 10.1186/s12931-024-02813-2 (PMC11046914; doi:10.1186/s12931-024-02813-2)
Supplement: Supplementary file 1 — Additional file 1: table S1. Manufacturer and catalog of antibodies. [file 12931_2024_2813_MOESM1_ESM.docx]

**Additional file 1 Table S1: Manufacturer and catalog of antibodies.**

| **Antibody** | **Manufacturer** | **Country** | **Catalog** |
| --- | --- | --- | --- |
| β-actin | ABclonal | China | AC028 |
| PCNA | ABclonal | China | A12427 |
| α-SMA | CST | USA | #19245 |
| SM22α | Huabio | China | ER62491 |
| α-tubulin | Huabio | China | ER130905 |
| β-tubulin | ABclonal | China | A12289 |
| MPO | Abcam | UK | ab208670 |
| PADI4 | Proteintech | USA | 17373-1-AP |
| CCDC25 | Biobyt | UK | orb446047 |
| ILK | Abcam | UK | ab52480 |
| β-parvin | Biorbyt | UK | orb637423 |
| RAC1 | Proteintech | USA | 66122-1-Ig |
| HRP Goat Anti-Rabbit IgG | ABclonal | China | AS014 |
| HRP Goat Anti-Mouse IgG | ABclonal | China | AS003 |
