# Supplementary figures and images for: Neutrophil extracellular traps promote proliferation of pulmonary smooth muscle cells mediated by CCDC25 in pulmonary arterial hypertension
Source: Respir Res. 2024 Apr 25;25:183. doi: 10.1186/s12931-024-02813-2 (PMC11046914; doi:10.1186/s12931-024-02813-2)

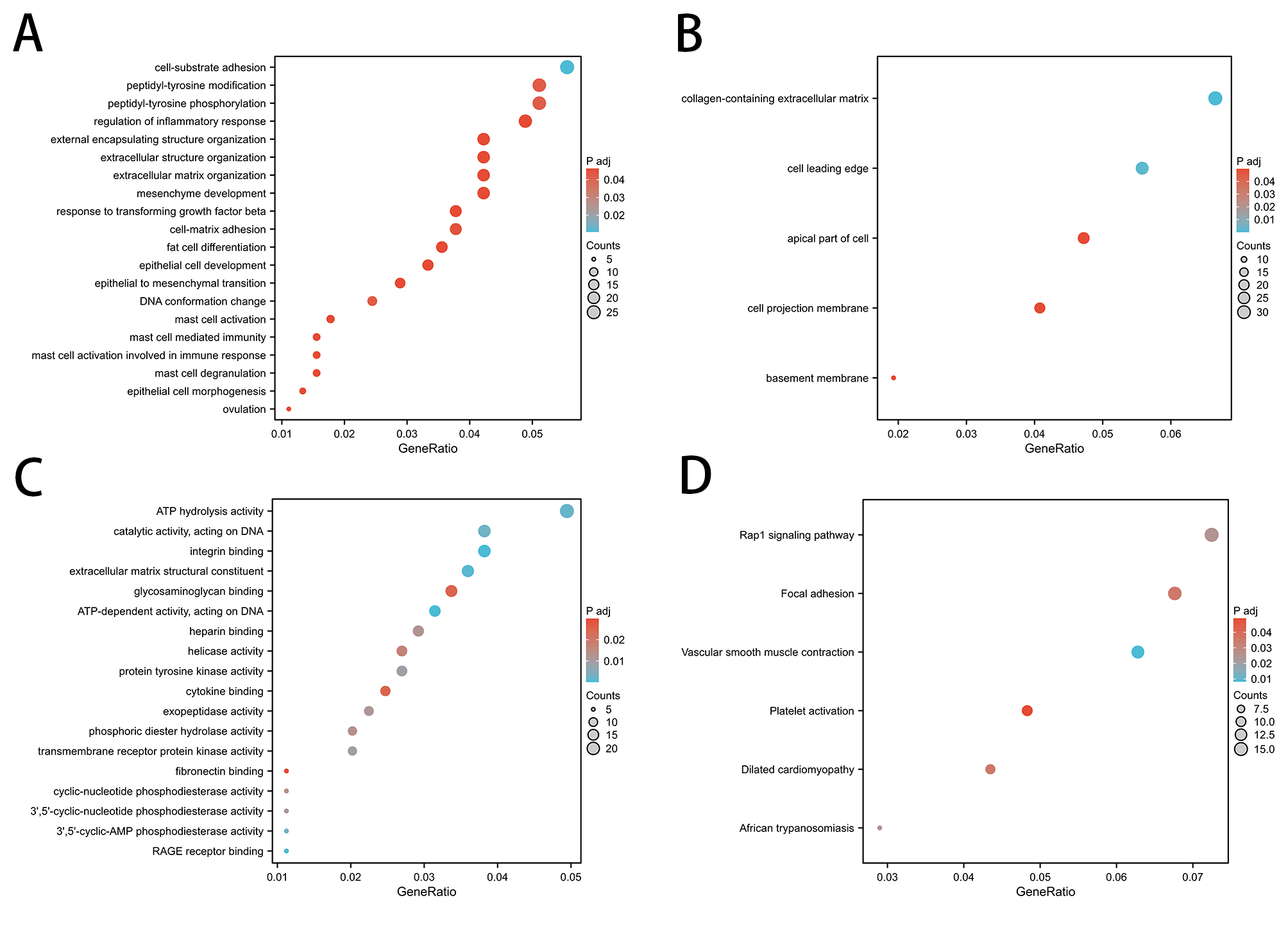

Supplement: Supplementary file 2 — Additional file 2: fig. S1. GO and KEGG enrichment analysis for microarray data. (A-C) GO enrichment analysis results. (D) KEGG enrichment analysis results. [file 12931_2024_2813_MOESM2_ESM.tif]

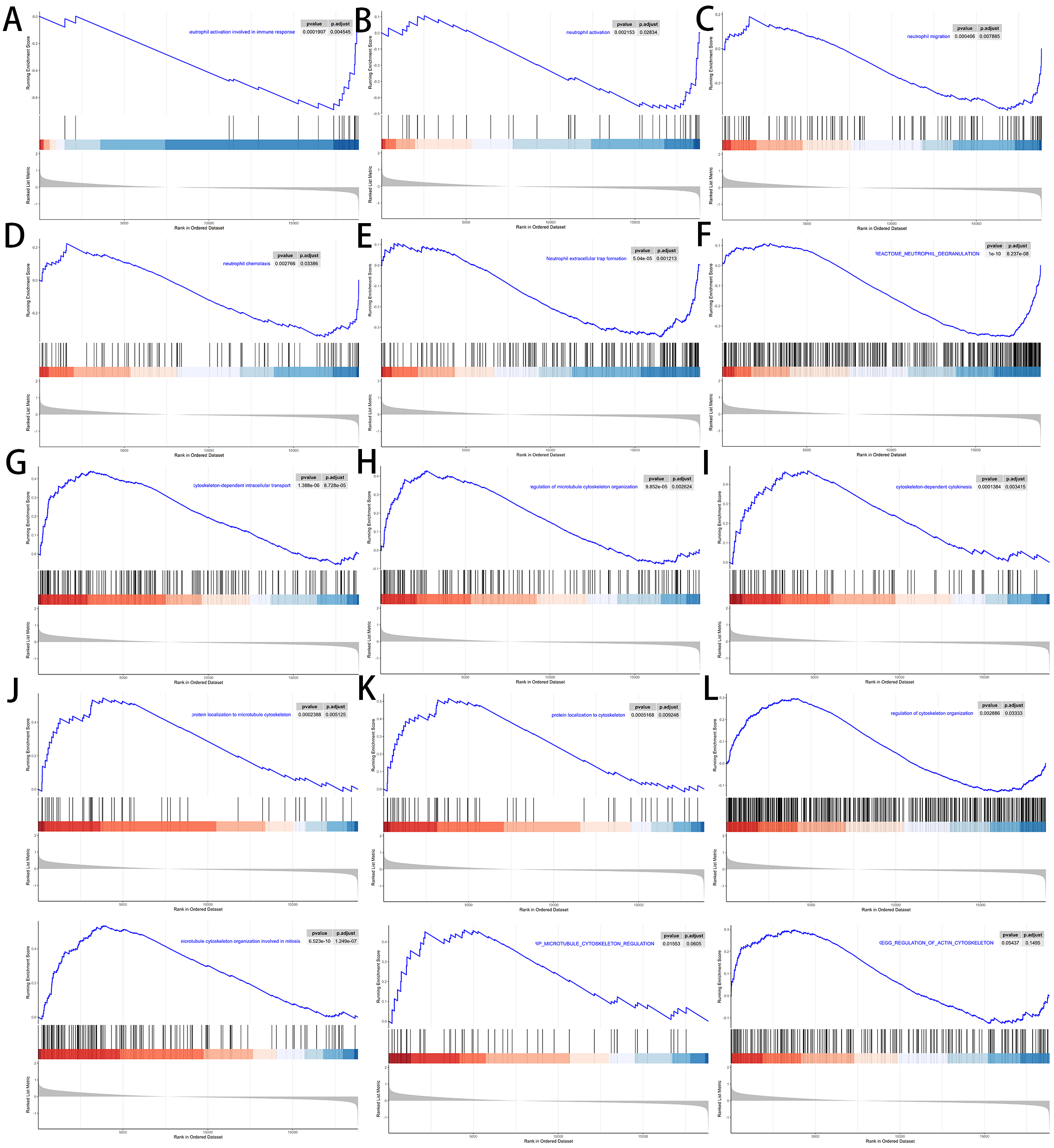

Supplement: Supplementary file 3 — Additional file 3: fig. S2. GSEA for microarray data. [file 12931_2024_2813_MOESM3_ESM.tif]

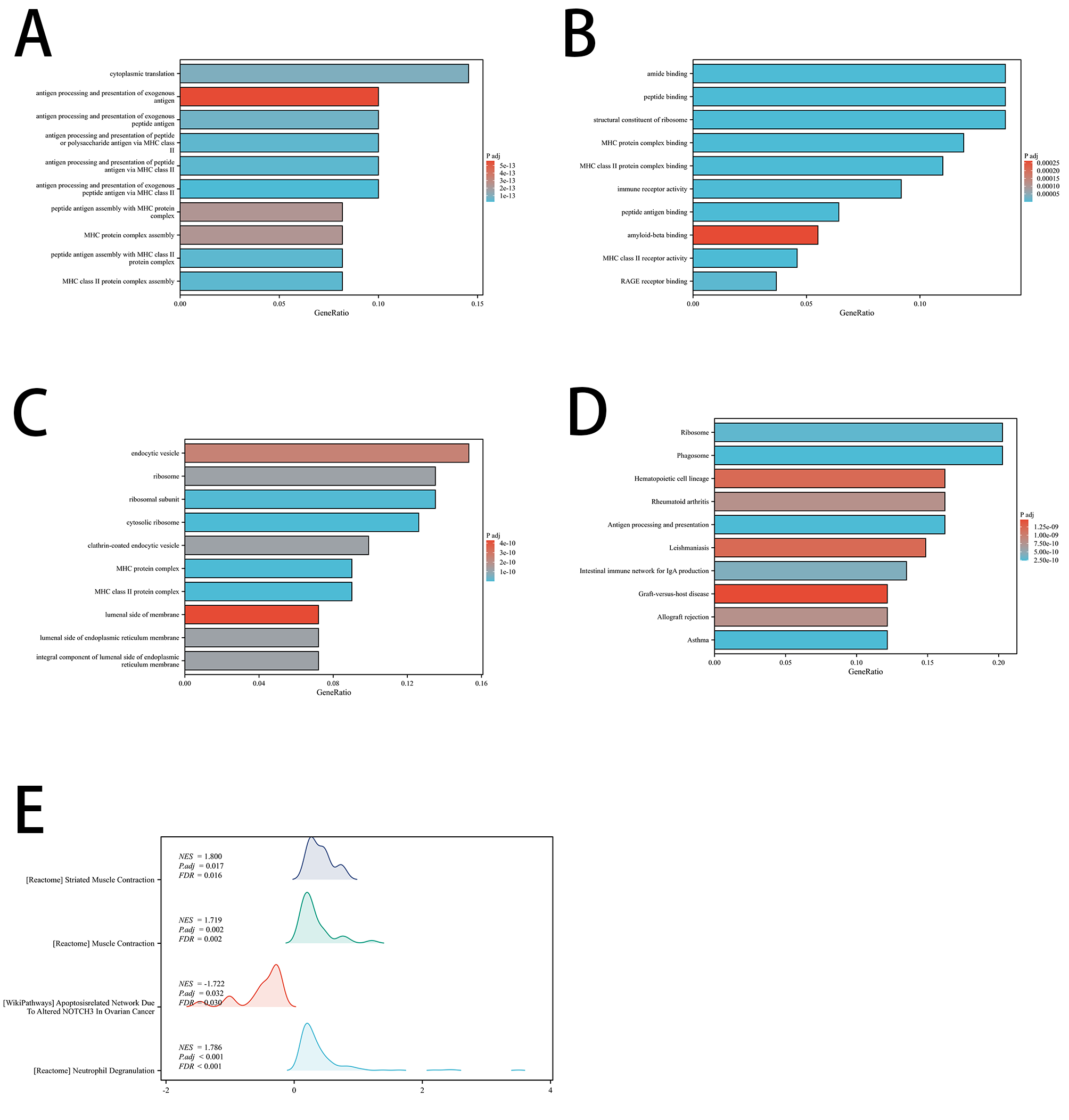

Supplement: Supplementary file 4 — Additional file 4: fig. S3. Enrichment analysis and GSEA for neutrophil single-cell sequencing data. (A-C) GO enrichment analysis results for neutrophil. (D) KEGG enrichment analysis results for neutrophil. (E) GSEA results for neutrophil. [file 12931_2024_2813_MOESM4_ESM.tif]

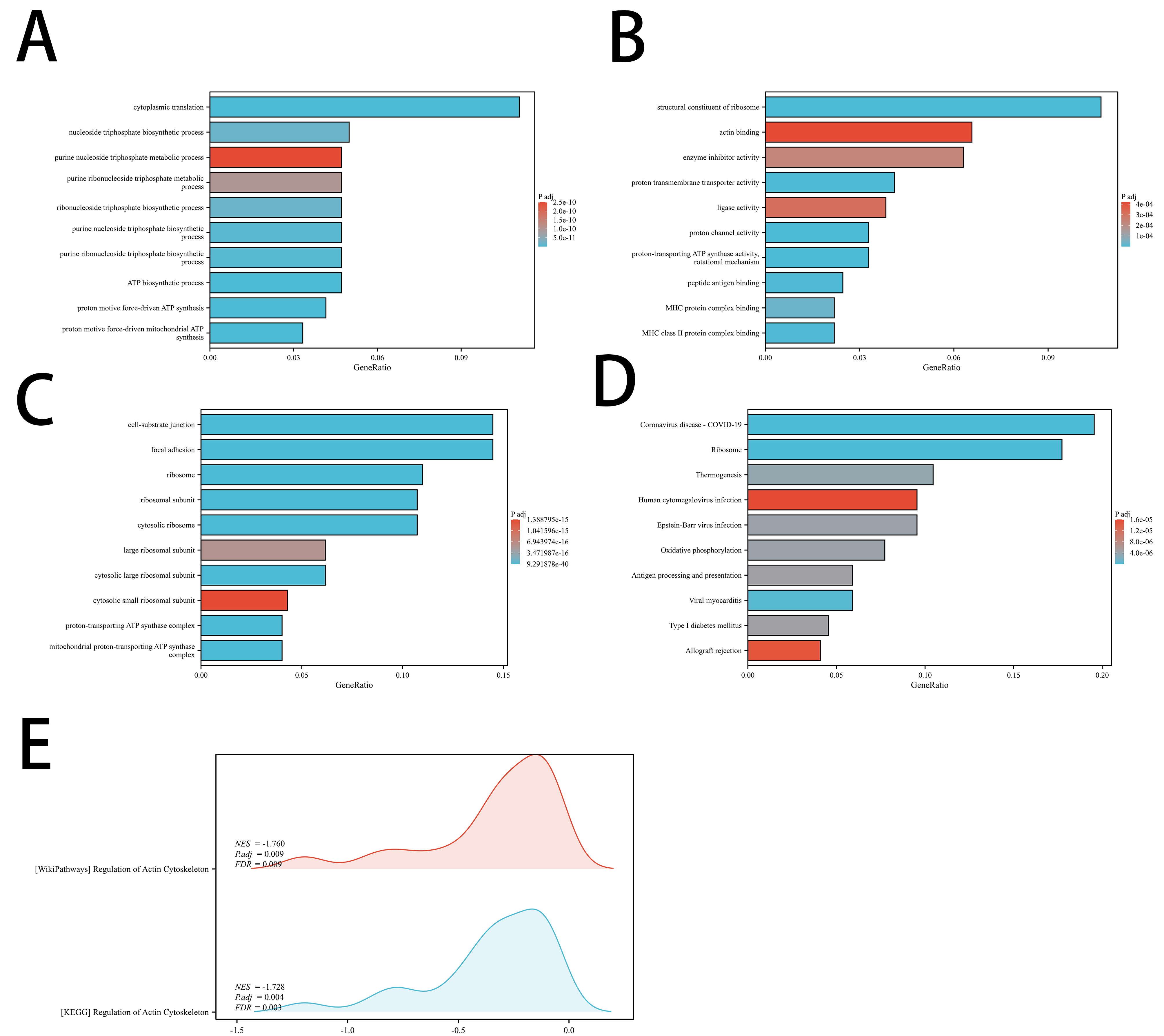

Supplement: Supplementary file 5 — Additional file 5: fig. S4. Enrichment analysis and GSEA for PASMCs single-cell sequencing data. (A-C) GO enrichment analysis results for PASMCs. (D) KEGG enrichment analysis results for PASMCs. (E) GSEA results for PASMCs. [file 12931_2024_2813_MOESM5_ESM.tif]
