# Supplementary material for: Neutrophil extracellular traps promote proliferation of pulmonary smooth muscle cells mediated by CCDC25 in pulmonary arterial hypertension
Source: Respir Res. 2024 Apr 25;25:183. doi: 10.1186/s12931-024-02813-2 (PMC11046914; doi:10.1186/s12931-024-02813-2)
Supplement: Supplementary file 6 — Supplementary Material 6 [file 12931_2024_2813_MOESM6_ESM.pdf]

Fig. 2J

PCNA

Target Protein

Control Protein

Repeat-1

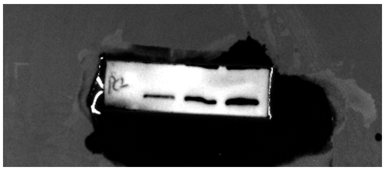

36 kDa

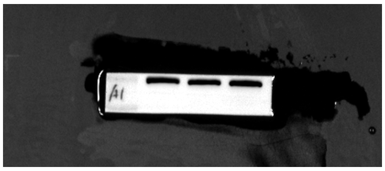

42 kDa

Repeat-2

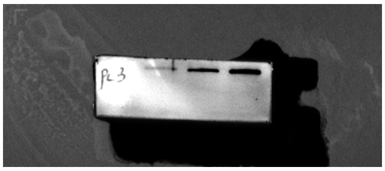

36 kDa

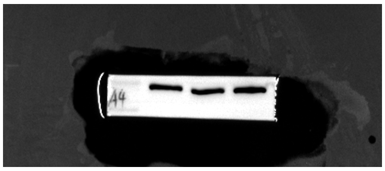

42 kDa

Repeat-3

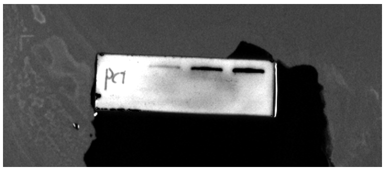

36 kDa

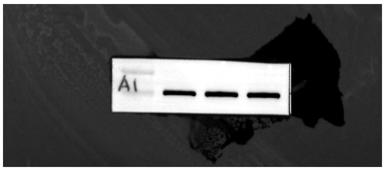

42 kDa

Fig. 3A

α-tubulin

Target Protein

Control Protein

Repeat-1

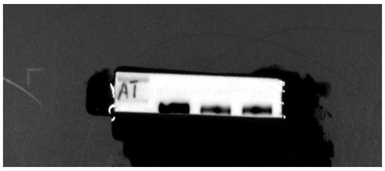

55 kDa

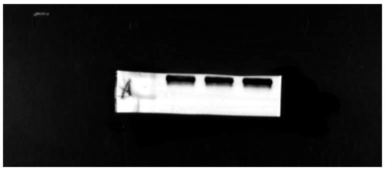

42 kDa

Repeat-2

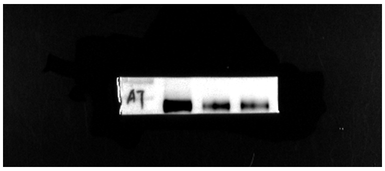

55 kDa

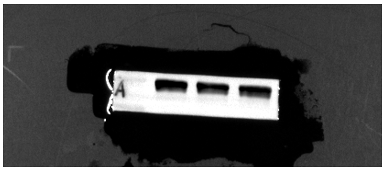

42 kDa

Repeat-3

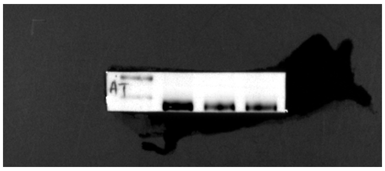

55 kDa

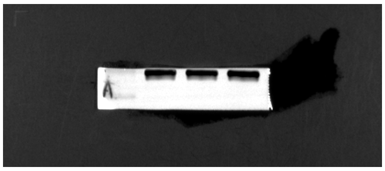

42 kDa

Fig. 3A

β-tubulin

Target Protein | Control Protein

Repeat-1
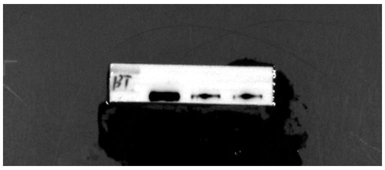
50 kDa
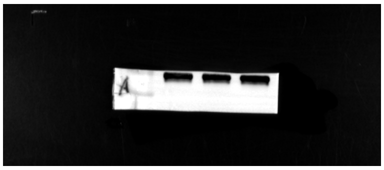
42 kDa

Repeat-2
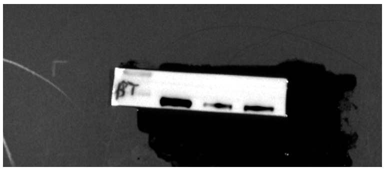
50 kDa
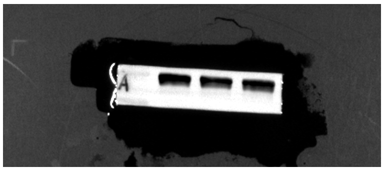
42 kDa

Repeat-3
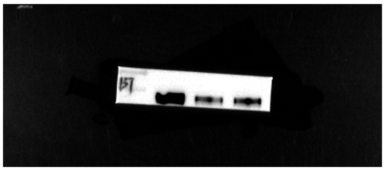
50 kDa
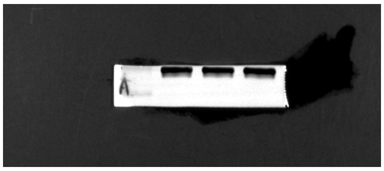
42 kDa

Fig. 3A

α-SMA

Target Protein | Control Protein

Repeat-1
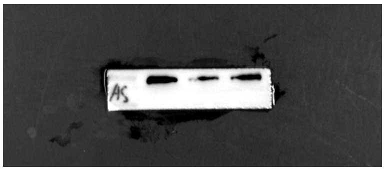
42 kDa
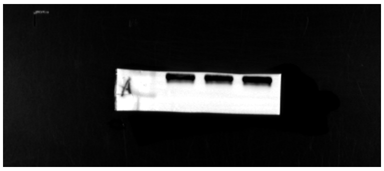
42 kDa

Repeat-2
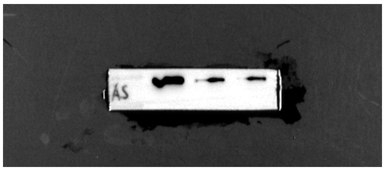
42 kDa
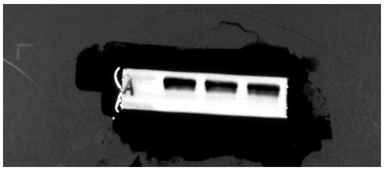
42 kDa

Repeat-3
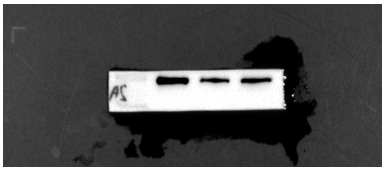
42 kDa
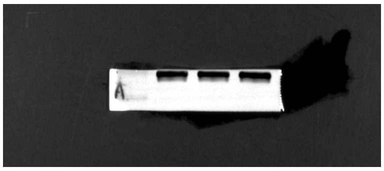
42 kDa

Fig. 3A

SM22α

Target Protein | Control Protein

Repeat-1 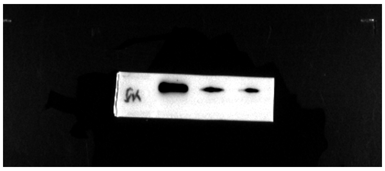 22 kDa 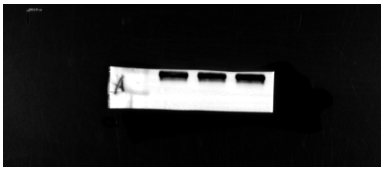 42 kDa

Repeat-2 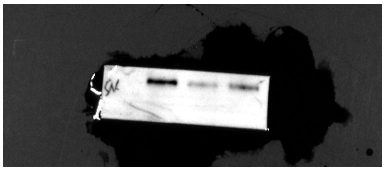 22 kDa 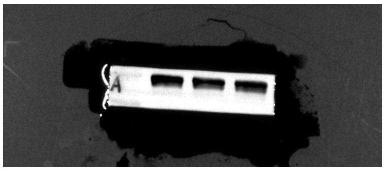 42 kDa

Repeat-3 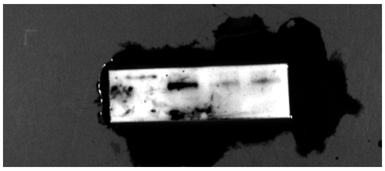 22 kDa 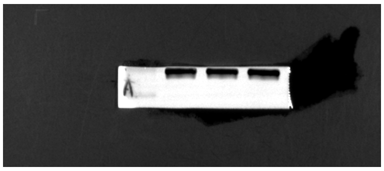 42 kDa

Fig. 3D

CCDC25

Target Protein | Control Protein

Repeat-1 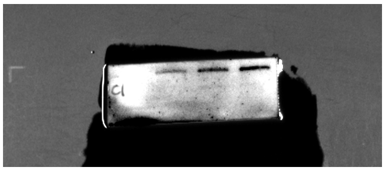 24 kDa 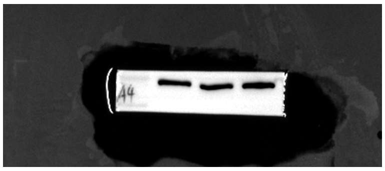 42 kDa

Repeat-2 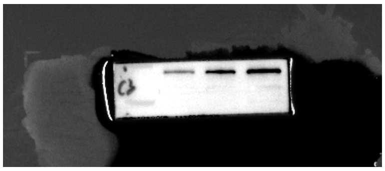 24 kDa 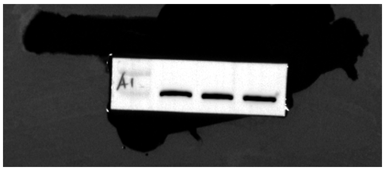 42 kDa

Repeat-3 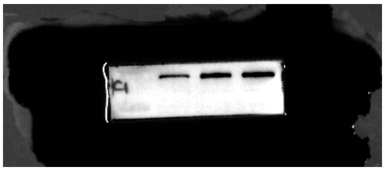 24 kDa 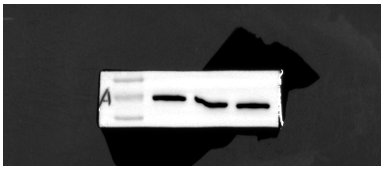 42 kDa

Fig. 3D

ILK

Target Protein

Control Protein

Repeat-1 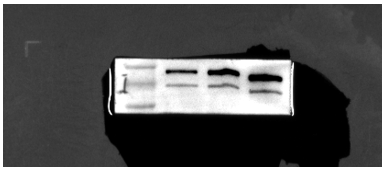 51 kDa 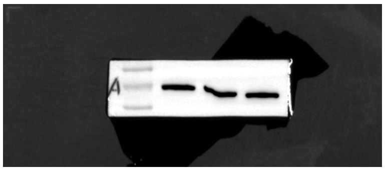 42 kDa

Repeat-2 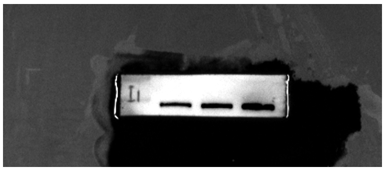 51 kDa 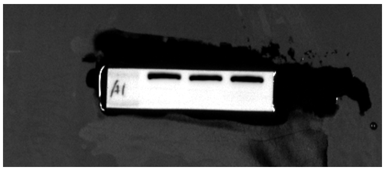 42 kDa

Repeat-3 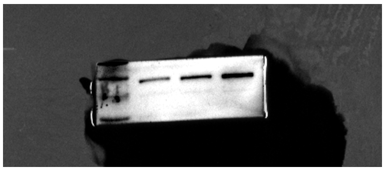 51 kDa 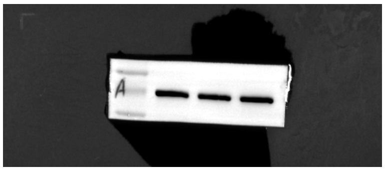 42 kDa

Fig. 3D

β-parvin

Target Protein

Control Protein

Repeat-1 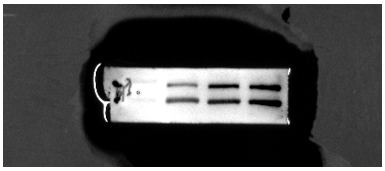 45 kDa 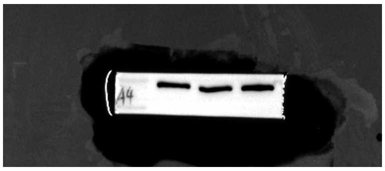 42 kDa

Repeat-2 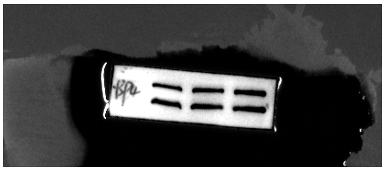 45 kDa 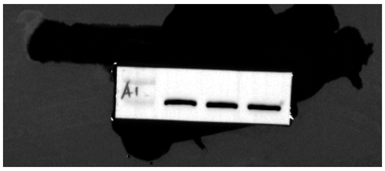 42 kDa

Repeat-3 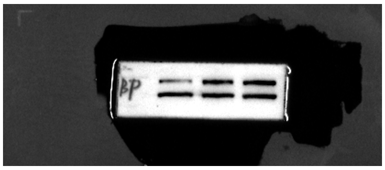 24 kDa 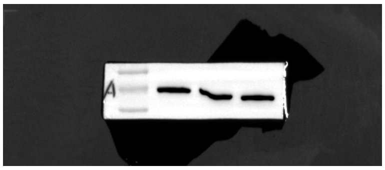 42 kDa

Fig. 3D

RAC1

Target Protein | Control Protein

Repeat-1
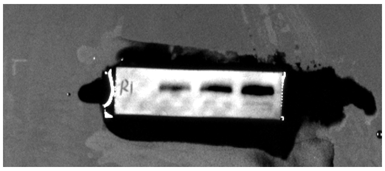
22 kDa
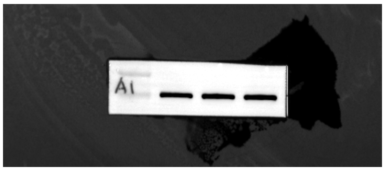
42 kDa

Repeat-2
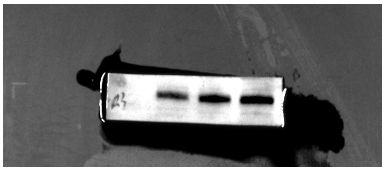
22 kDa
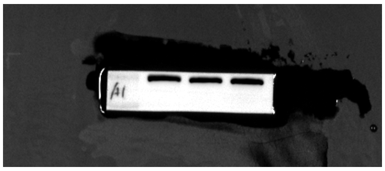
42 kDa

Repeat-3
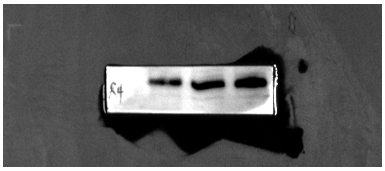
22 kDa
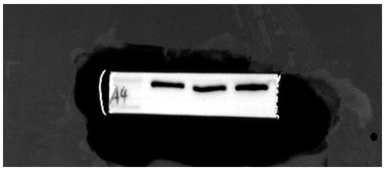
42 kDa

Fig. 4E

PCNA

Repeat-1

Target Protein
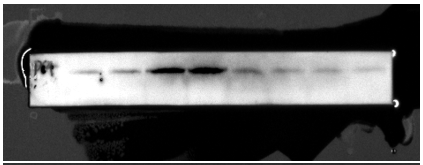
36 kDa

Control Protein
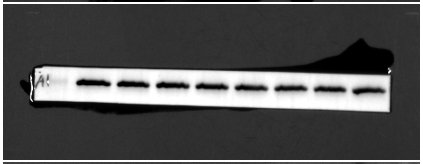
42 kDa

Repeat-2

Target Protein
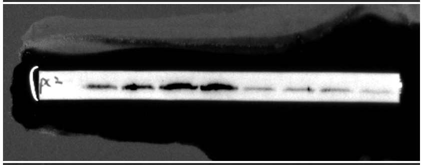
36 kDa

Control Protein
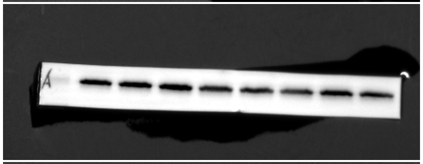
42 kDa

Repeat-3

Target Protein
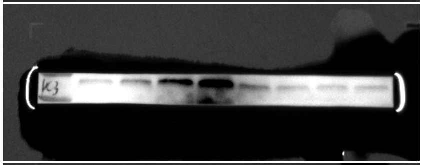
36 kDa

Control Protein
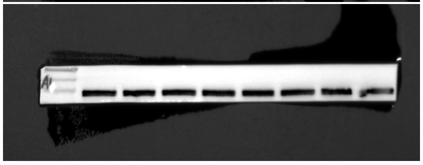
42 kDa

Fig. 4G

PADI4

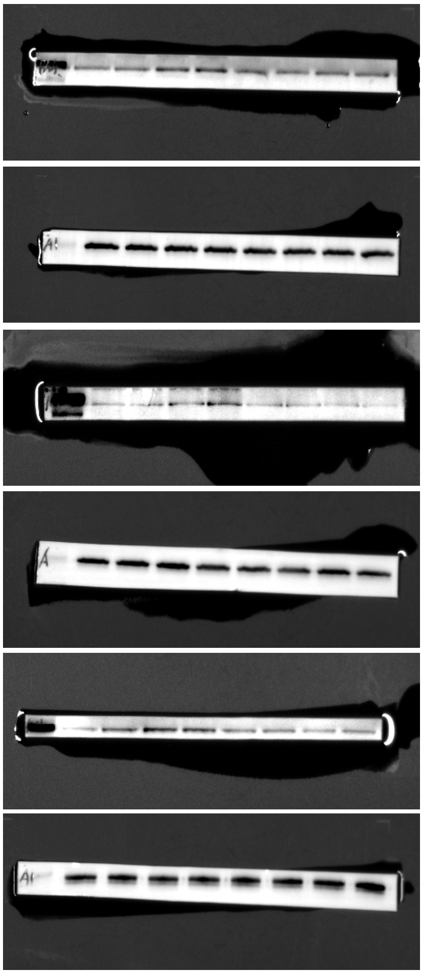

Repeat-1

Target Protein 67 kDa

Control Protein 42 kDa

Repeat-2

Target Protein 67 kDa

Control Protein 42 kDa

Repeat-3

Target Protein 67 kDa

Control Protein 42 kDa

Fig. 4G

MPO

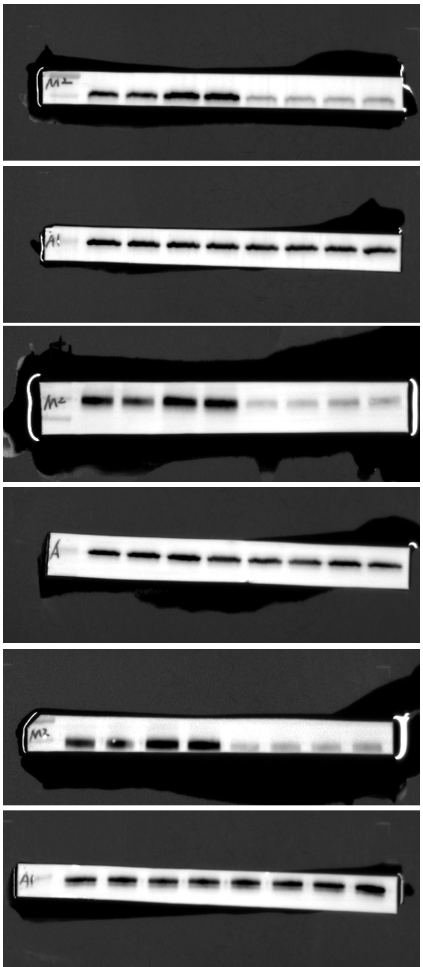

Repeat-1

Target Protein 59 kDa

Control Protein 42 kDa

Repeat-2

Target Protein 59 kDa

Control Protein 42 kDa

Repeat-3

Target Protein 59 kDa

Control Protein 42 kDa

Fig. 4I

CCDC25

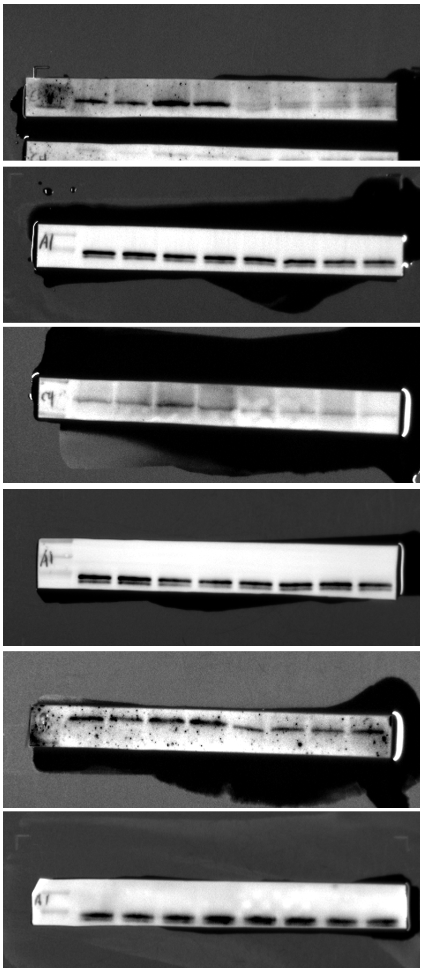

Repeat-1
Target Protein 24 kDa
Control Protein 42 kDa

Repeat-2
Target Protein 24 kDa
Control Protein 42 kDa

Repeat-3
Target Protein 24 kDa
Control Protein 42 kDa

Fig. 4I

ILK

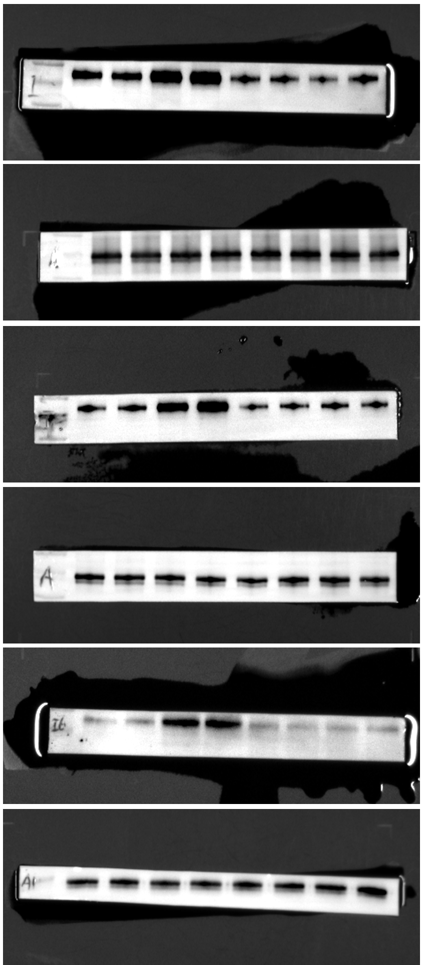

Repeat-1
Target Protein 51 kDa
Control Protein 42 kDa

Repeat-2
Target Protein 51 kDa
Control Protein 42 kDa

Repeat-3
Target Protein 51 kDa
Control Protein 42 kDa

Fig. 4I

β-parvin

Repeat-1

Target Protein 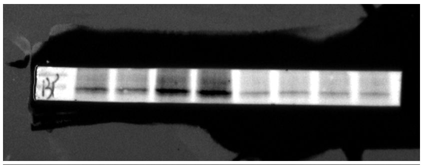 45 kDa

Control Protein 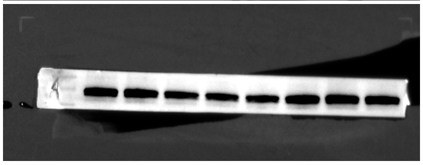 42 kDa

Repeat-2

Target Protein 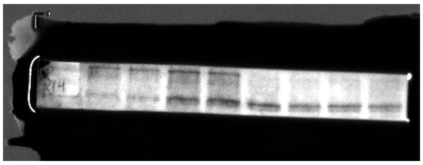 45 kDa

Control Protein 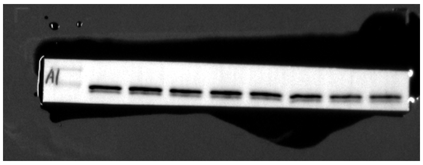 42 kDa

Repeat-3

Target Protein 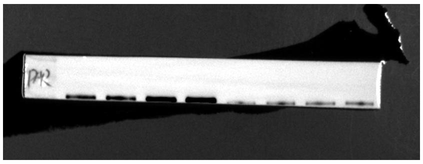 45 kDa

Control Protein 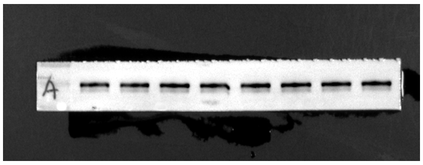 42 kDa

Fig. 4I

RAC1

Repeat-1

Target Protein 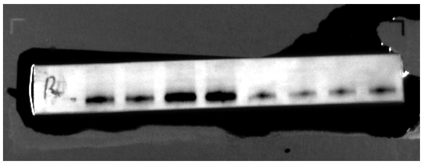 21 kDa

Control Protein 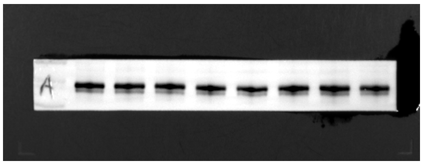 42 kDa

Repeat-2

Target Protein 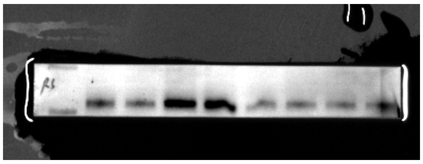 21 kDa

Control Protein 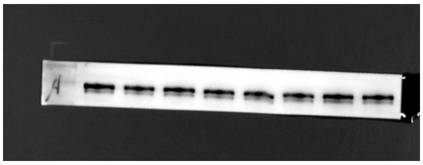 42 kDa

Repeat-3

Target Protein 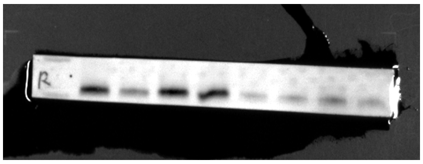 21 kDa

Control Protein 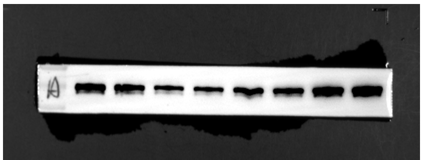 42 kDa

Fig. 5F

PCNA

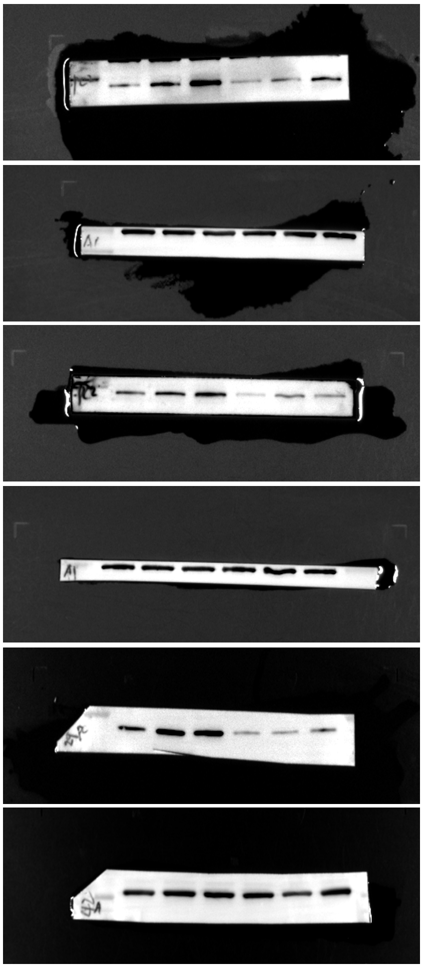

Repeat-1

Target Protein 36 kDa

Control Protein 42 kDa

Repeat-2

Target Protein 36 kDa

Control Protein 42 kDa

Repeat-3

Target Protein 36 kDa

Control Protein 42 kDa

Fig. 6A

α-tubulin

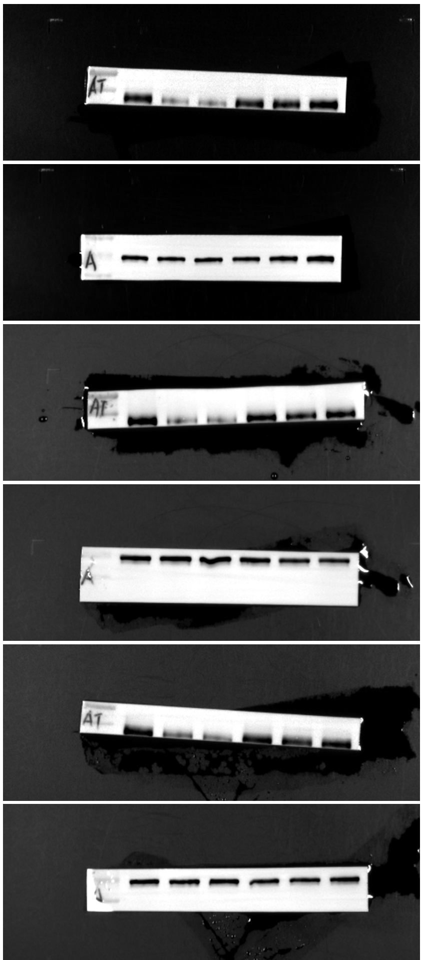

Repeat-1

Target Protein 55 kDa

Control Protein 42 kDa

Repeat-2

Target Protein 55 kDa

Control Protein 42 kDa

Repeat-3

Target Protein 55 kDa

Control Protein 42 kDa

Fig. 6A

β-tubulin

Repeat-1

Target Protein

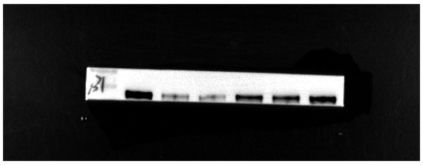

50 kDa

Control Protein

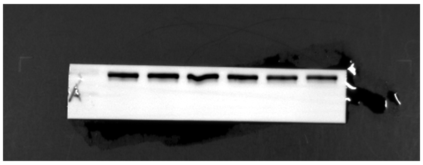

42 kDa

Repeat-2

Target Protein

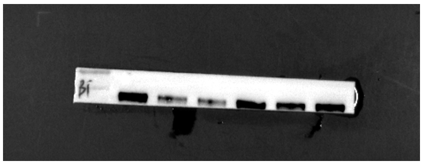

50 kDa

Control Protein

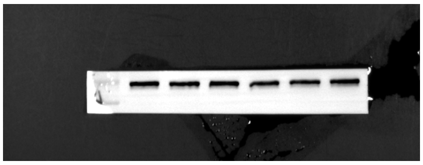

42 kDa

Repeat-3

Target Protein

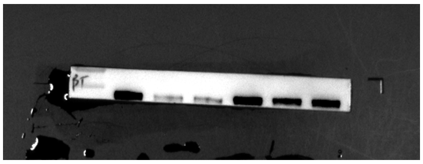

50 kDa

Control Protein

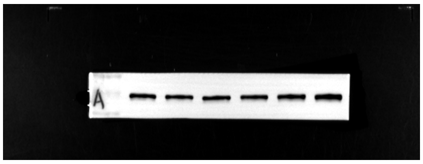

42 kDa

Fig. 6A

α-SMA

Repeat-1

Target Protein

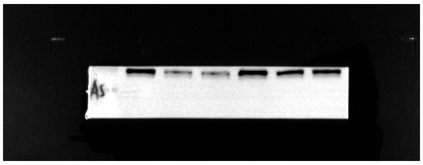

42 kDa

Control Protein

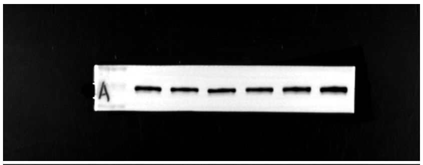

42 kDa

Repeat-2

Target Protein

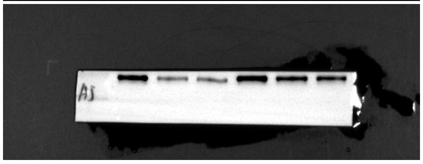

42 kDa

Control Protein

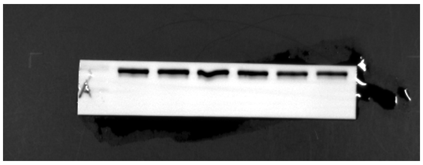

42 kDa

Repeat-3

Target Protein

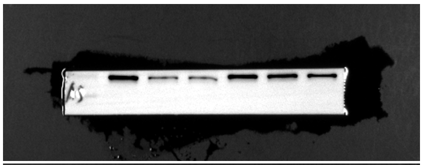

42 kDa

Control Protein

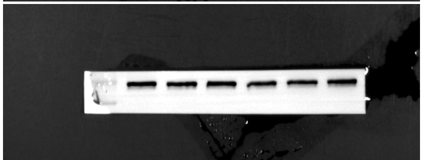

42 kDa

Fig. 6A

SM22α

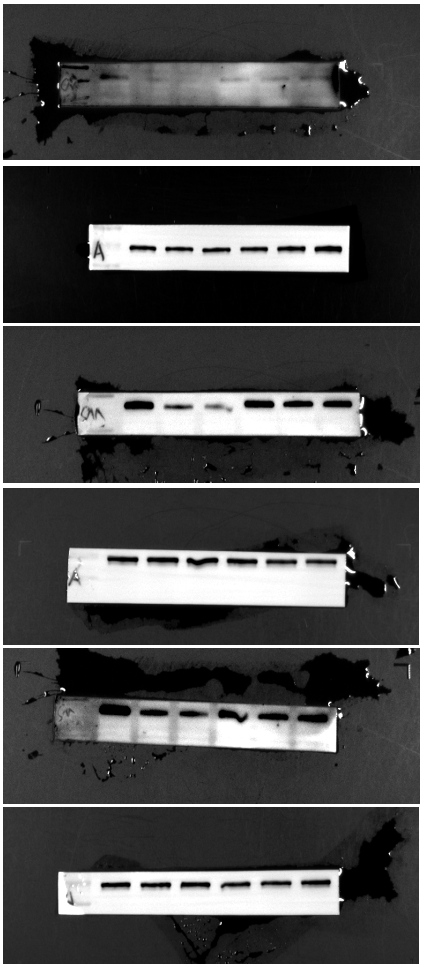

Repeat-1
Target Protein 22 kDa
Control Protein 42 kDa

Repeat-2
Target Protein 22 kDa
Control Protein 42 kDa

Repeat-3
Target Protein 22 kDa
Control Protein 42 kDa

Fig. 6D

CCDC25

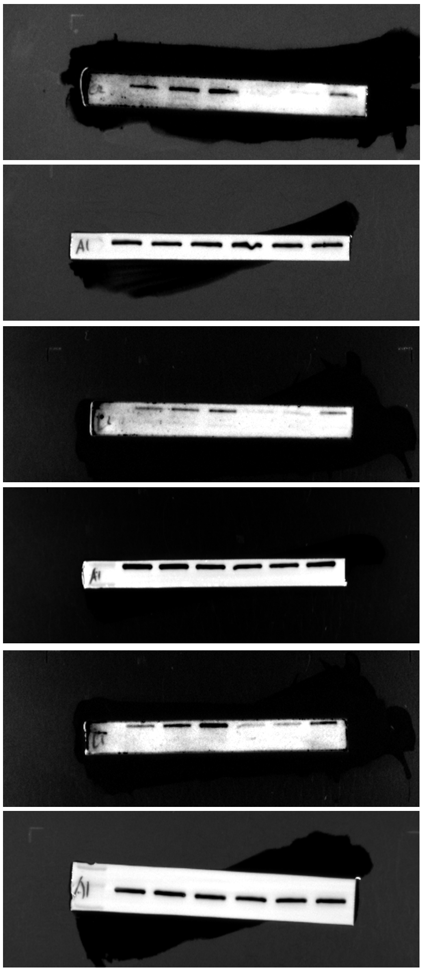

Repeat-1
Target Protein 24 kDa
Control Protein 42 kDa

Repeat-2
Target Protein 24 kDa
Control Protein 42 kDa

Repeat-3
Target Protein 24 kDa
Control Protein 42 kDa

Fig. 6D

ILK

Target Protein 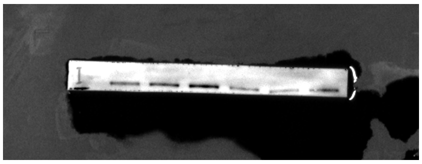 51 kDa

Repeat-1

Control Protein 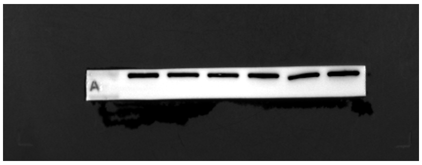 42 kDa

Target Protein 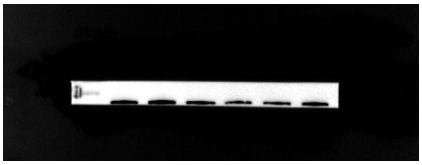 51 kDa

Repeat-2

Control Protein 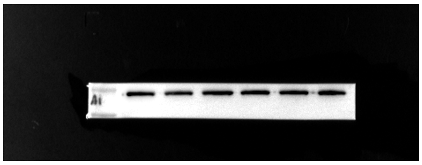 42 kDa

Target Protein 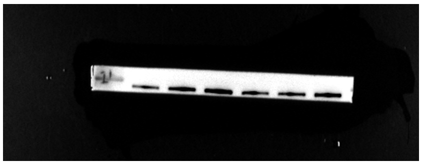 51 kDa

Repeat-3

Control Protein 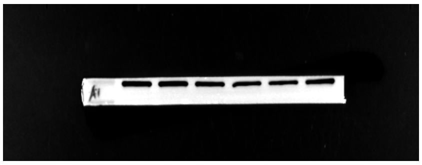 42 kDa

Fig. 6D

β-parvin

Target Protein 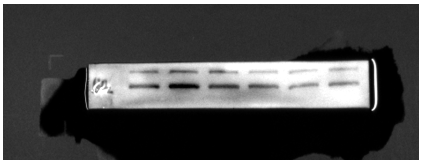 45 kDa

Repeat-1

Control Protein 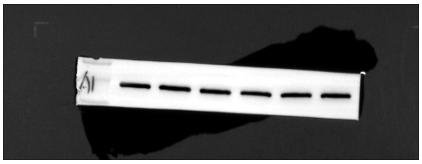 42 kDa

Target Protein 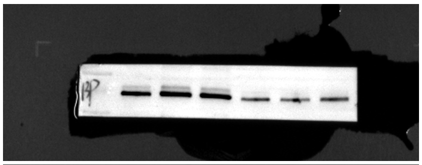 45 kDa

Repeat-2

Control Protein 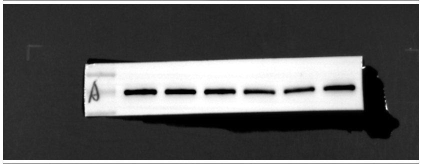 42 kDa

Target Protein 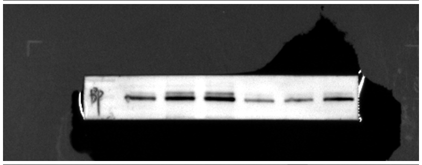 45 kDa

Repeat-3

Control Protein 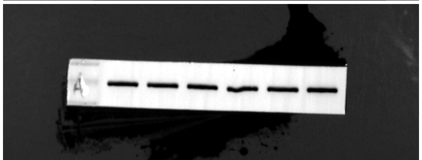 42 kDa

Fig. 6D

RAC1

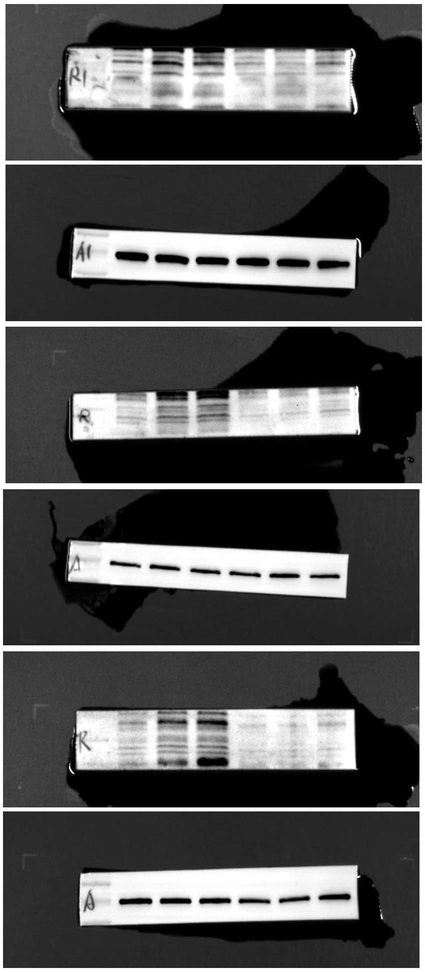

Repeat-1

Target Protein 21 kDa

Control Protein 42 kDa

Repeat-2

Target Protein 21 kDa

Control Protein 42 kDa

Repeat-3

Target Protein 21 kDa

Control Protein 42 kDa
